# Transcriptomic analysis identifies candidate molecular mechanisms underlying SCF+G-CSF-reduced neuropathology in aged APP/PS1 mice

**DOI:** 10.3389/fnagi.2026.1705436

**Published:** 2026-10-06

**Authors:** Afua Addo, Bin Li, Sasidhar Murikinati, Robert Gardner, Li-Ru Zhao

**Affiliations:** 1 Department of Neurosurgery, Norton College of Medicine, SUNY Upstate Medical University, Syracuse, NY, United States; 2 Department of Neurology, Louisiana State University Health Sciences Center, Shreveport, LA, United States

**Keywords:** Alzheimer’s disease, granulocyte colony-stimulating factor, hematopoietic growth factors, immunomodulation, stem cell factor, transcriptomics

## Abstract

**Background:**

Alzheimer’s Disease (AD) is a neurodegenerative disease characterized by amyloid plaque deposition, tau hyperphosphorylation, neuroinflammation, and cognitive decline. Our previous studies demonstrated that combined treatment with stem cell factor (SCF) and granulocyte colony-stimulating factor (G-CSF) reduces AD pathology in APP/PS1 mice. The present study aimed to explore the molecular mechanisms underlying the effects of SCF+G-CSF using transcriptomic analysis.

**Methods:**

Aged APP/PS1 mice received daily subcutaneous injections of SCF+G-CSF or vehicle for 12 days. RNA was extracted from brain tissue on day 13 for gene-chip analysis. Age-matched wild-type (WT) mice served as controls. Data were analyzed using TAC, STRING v12.0, Reactome, and ShinyGO 0.77. A total of 45,037 genes were analyzed. Twenty-seven differentially expressed genes (DEGs) met a ≥ 2-fold threshold in SCF+G-CSF-treated versus vehicle-treated APP/PS1 mice, whereas 89 DEGs met this threshold in APP/PS1 versus WT mice.

**Results:**

SCF+G-CSF treatment upregulated immune-related genes, including *S100a8, S100a9, Ngp, Lcn2, Ltf*, and *Camp*, which are associated with immune regulation and amyloid-related processes. Pathway analysis identified downregulation of IL-2, IL-4, IL-7, and EGFR1 signaling and enrichment of IL-17 signaling, suggesting modulation of both innate and adaptive immune responses. SCF+G-CSF also downregulated several genes with known oncogenic functions, including *Cbl, Akap9, Kcnq1ot1*, and *Snhg11*, suggesting a potential molecular overlap between cancer- and AD-related pathways. In addition, pathway enrichment analysis identified NADPH oxidase activation through Rho GTPase signaling and greater than 400-fold enrichment of metal ion sequestration pathways following SCF+G-CSF treatment.

**Conclusion:**

SCF+G-CSF treatment was associated with broad transcriptional changes involving immune regulation, metal ion homeostasis, and genes with known oncogenic functions. These findings provide new insights into the molecular pathways influenced by SCF+G-CSF in aged APP/PS1 mice and identify candidate mechanisms and therapeutic targets for future investigation.

## 1 Introduction

Alzheimer’s Disease (AD) is a progressive neurodegenerative disorder characterized by extracellular amyloid beta (Aβ) plaque accumulation, intracellular tau hyperphosphorylation leading to neurofibrillary tangle (NFT) formation, activation of innate immune cells, neuroinflammation, and cognitive decline (Long and Holtzman, 2019; Rajesh and Kanneganti, 2022). Despite numerous efforts to target these hallmarks, AD remains the leading cause of dementia, projected to affect nearly 14 million Americans by 2060 (Alzheimer’s Association, 2025), with costs of care exceeding $300 billion in 2020 (Wong, 2020).

Non-pharmacological interventions, such as physical exercise and cognitive training, have been shown to improve behavioral symptoms (Wong, 2020). However, the efficacy of current pharmacological treatments remains limited. FDA-approved cholinesterase inhibitors, NMDA receptor modulators, monoclonal antibodies targeting Aβ, NSAIDs, and cholesterol-lowering agents have largely failed to stop disease progression or demonstrate consistent clinical benefit in mouse models or AD patients (Long and Holtzman, 2019; Potter et al., 2021).

These outcomes underscore the need for new therapeutic approaches. Increasing attention has turned to the roles of innate and adaptive immune responses and neuroinflammation in AD pathogenesis. Microglia, the resident macrophages of the central nervous system (CNS), play a critical role in clearing Aβ and cellular debris and regulating neuroinflammatory signaling (Lampron et al., 2011; Long and Holtzman, 2019; Rajesh and Kanneganti, 2022). Upon detecting pathological changes via pattern-recognition receptors, microglia and other CNS immune cells, such as astrocytes, initiate inflammatory cascades involving cytokines, including IL-1β, TNF-α, and IL-6 (Rajesh and Kanneganti, 2022). While inflammation aids in clearing debris, its chronic activation can worsen disease progression (Rajesh and Kanneganti, 2022).

In healthy brains, Aβ is efficiently cleared by microglia; however, this process becomes progressively impaired during AD progression (Rajesh and Kanneganti, 2022). In addition, multiple forms of programmed cell death, including apoptosis, pyroptosis, and necroptosis, have been implicated in chronic neuroinflammation, contributing to tau misfolding, disruption of tissue homeostasis, and impaired clearance of cellular debris (Rajesh and Kanneganti, 2022). It has been reported that peripherally derived monocytes infiltrate the brain parenchyma, target amyloid plaques, and contribute to reducing amyloid plaque burden in aged APP/PS1 mice (Yan et al., 2022). Furthermore, splenectomy reduced the abundance of plaque-associated macrophages and increased amyloid plaque burden, further supporting a protective role for peripheral monocytes/macrophages in Aβ clearance (Yan et al., 2022). Collectively, these findings highlight the important roles of innate immune cells, including resident microglia and peripherally derived monocytes/macrophages, in regulating neuroinflammation and Aβ clearance, making these processes attractive therapeutic targets for AD.

Beyond debris clearance, glial cells also mediate chemoattraction, recruitment, and activation of immune cells (Long and Holtzman, 2019; Rajesh and Kanneganti, 2022). This highlights the potential of modulating intrinsic glial functions and hematopoiesis, the process of generating blood and immune cells (Lampron et al., 2011), as a therapeutic avenue. Although hematopoietic system dysfunction is implicated in AD, its therapeutic targeting remains underexplored (Lampron et al., 2011). Hematopoietic growth factors such as stem cell factor (SCF) and granulocyte colony-stimulating factor (G-CSF) regulate the proliferation, survival, migration, and differentiation of hematopoietic stem cells (HSCs) (Demetri and Griffin, 1991; Galli et al., 1994; McNiece and Briddell, 1995).

Clinical studies reveal that rheumatoid arthritis patients exhibit a lower AD risk (Policicchio et al., 2017), potentially due to elevated circulating hematopoietic growth factors such as G-CSF and GM-CSF levels (Nakamura et al., 2000; Olszewski et al., 2001). Recombinant GM-CSF (Sargramostim) has demonstrated preliminary clinical evidence of immune modulation and cognitive benefit in AD patients (Potter et al., 2021).

Our previous studies demonstrated that combined treatment with hematopoietic growth factors, SCF and G-CSF (SCF+G-CSF), significantly reduced Aβ burden and neuroinflammation while enhancing synaptic and dendritic regeneration in middle-aged and aged APP/PS1 mice (Guo et al., 2020; Li et al., 2011). SCF+G-CSF-treated aged APP/PS1 mice exhibited reduced compact and fibrillary Aβ deposits, decreased expression of pro-inflammatory markers, including NOS, increased anti-inflammatory IL-4 expression, and elevated levels of TREM2, a microglial receptor essential for amyloid clearance (Guo et al., 2020). Despite these findings, the molecular mechanisms underlying the therapeutic effects of SCF+G-CSF remain incompletely understood. Therefore, the objective of the present study was to use transcriptomic analysis to identify treatment-induced transcriptional changes and biological pathways that may provide mechanistic insight into the therapeutic effects of SCF+G-CSF in aged APP/PS1 mice.

## 2 Materials and methods

All animal procedures were conducted in accordance with the National Institute of Health Guide for the Care and Use of Laboratory Animals and were approved by the Institutional Animal Care and Use Committee.

### 2.1 Animals and experimental design

APPswe/PS1dE9 (APP/PS1) mice (stock# 34832) and C57BL/6J mice (Wild Type control mice) (stock# 000664) (The Jackson Laboratory) were used in this study. The APP/PS1 mice co-express Swedish double mutations (K595N/M596L) of amyloid precursor protein (APP) and mutant human presenilin protein 1 (PS1).

Our previous studies showed that 12-day treatment with SCF+G-CSF (s.c.) mitigated AD neuropathology in both middle-aged and aged APP/PS1 mice (Guo et al., 2020; Li et al., 2011); therefore, this study followed the same treatment paradigm to explore the mechanism of SCF+G-CSF treatment in aged APP/PS1 mice with advanced AD neuropathology using gene chip technology.

APP/PS1 mice (male, 17–18 months old) were randomly divided into two groups: a vehicle control group (*n* = 3) and an SCF+G-CSF treatment group (*n* = 3). Age and sex-matched C57BL/6J mice served as wild-type (WT) controls (*n* = 2). Recombinant mouse SCF (200 mg/kg/day; PeproTech, United States) and recombinant human G-CSF (50 mg/kg/day; Amgen, United States) or an equal volume of saline were subcutaneously (s.c.) injected for 12 consecutive days. One day (day 13) after the final injection, mice were anesthetized with sodium pentobarbital (50 mg/kg, i.p.) and transcardially perfused with heparinized phosphate-buffered saline (PBS, 10 U/mL) to remove blood. Brains were then rapidly removed, and brain tissue from the frontal lobes was dissected for RNA extraction and used for gene chip analysis. Following dissection, brain tissue was rapidly frozen on dry ice and stored at −80°C until further processing.

The present study used recombinant human G-CSF (rhG-CSF) in a murine model. Although species-specific immune responses cannot be completely excluded, the functional cross-reactivity of rhG-CSF in mice is well established. Recombinant human G-CSF has been shown to stimulate colony formation in both human and mouse bone marrow cells (Tsuchiya et al., 1987); activate conserved signaling pathways, including JAK, STAT3, and Akt, through the murine G-CSF receptor (Park et al., 2022); and promote hematopoietic stem cell mobilization in peripheral blood and spleen of mice (Bungart et al., 1990; Drize et al., 1993). Together, these studies demonstrate the biological activity of rhG-CSF in murine systems. Importantly, previous murine research has reported the use of rhG-CSF at doses of up to 250 μg/kg/day for hematopoietic mobilization (Drize et al., 1993). In contrast, we intentionally used a relatively low dose (50 μg/kg/day) to minimize excessive immune stimulation while maintaining activation of conserved G-CSF receptor signaling pathways.

### 2.2 RNA extraction method

Frozen brain tissue was transferred into RNA*later* stabilization solution (Catalog# AM7021, ThermoFisher, United States) at a ratio of 100 mg of tissue per 500 ml of RNA*later*. RNA extraction was performed using the RNeasy Lipid Tissue Mini Kit (Catalog# 74804, Qiagen, United States). Briefly, after removing the brain tissue from RNA*later*, it was homogenized in 1 ml of QIAzolL Lysis Reagent using a glass homogenizer ( ≤ 100 mg brain tissue per sample).

The homogenate was incubated at room temperature (15–25°C) for 5 min, then centrifuged at 14,000 × g for 30 s. The resulting homogenate was transferred to a MaXtract tube along with 70 mL RNase-free water. Next, 200 mL of Chloroform was added, the tube was shaken vigorously for 15 seconds, incubated at room temperature for 2–3 min, and centrifuged at 12,000 × g for 15 min at 4°C.

The upper, aqueous phase was transferred to a new tube, mixed with 600 mL of 70% ethanol, and vortexed. The sample was then loaded onto a RNeasy Mimi spin column placed in a 2 ml collection tube and centrifuged at 8,000 × g for 15 s at room temperature. The flow-through was discarded.

To wash the membrane, 350 mL Buffer RW1 was added to the spin column and centrifuged at 8,000 × g for 15 s. The flow-through was discarded. A DNase digestion step was performed by mixing 10 mL of DNase I stock solution with 70 mL of Buffer RDD (Cat# 79254, RNase-Free DNase Set, Qiagen, United States). After gentle inversion, 80 mL of the DNase 1 mix was applied directly to the spin column membrane and incubated for 15 min at room temperature.

Following DNase digestion, 350 mL of Buffer RW1 was added to the spin column, centrifuged at 8,000 × g for 15 s, and the flow-through was discarded. The membrane was then washed with 500 mL of Buffer RPE, centrifuged at 8,000 × g for 15 s, and the flow-through was discarded. A second wash was performed using the same volume of Buffer RPE, followed by centrifugation at 8,000 × g for 2 min.

The spin column was transferred to a new 2 mL collection tube and centrifuged at full speed for 1 min to remove residual buffer. It was then placed in a new 1.5 mL collection tube. To elute RNA, 30 mL of RNase-free water was added directly to the column membrane and centrifuged at 8,000 × g for 1 min.

RNA integrity and purity were assessed using an Agilent Bioanalyzer before proceeding with gene chip analysis.

### 2.3 Gene chip data analysis

Transcriptomic analysis of the gene chip data was performed using ThermoFisher Scientific’s Transcriptome Analysis Console (TAC) (RRID:SCR_016519). “.cel” files containing the microarray data for mice labeled APP1, APP2, APP3, CON1, CON2, CON3, WT1, and WT2 were uploaded into TAC for gene expression comparison analysis after selecting the Mouse 430_2.0 array type, with the genome version set to “Mm10 (Mus musculus).” Two separate analyses were performed: (1) SCF+G-CSF-treated APP/PS1 mice (APP) versus vehicle-treated APP/PS1 control mice (CON) and (2) CON versus WT controls. The analysis type was set to “Expression (gene),” and RMA was selected for summarization. The one-way ANOVA empirical Bayes (“ebayes”) method was used for data analysis, and a 2.0-fold expression threshold was applied with a gene-level *p*-value cutoff of < 0.05. Out of the 45,037 genes detected from the microarray “.cel” files, 27 genes and 89 genes were significantly differentially expressed in the APP versus CON and CON versus WT comparisons, respectively.

The resulting differentially expressed gene lists were entered into publicly available gene analysis tools, including STRING v12.0 (Szklarczyk et al., 2023), ShinyGO v0.77 (Ge et al., 2020) (developed by South Dakota State University), and Reactome (Griss et al., 2020) for further analysis.

## 3 Results and discussion

Given that our previous studies demonstrated that SCF+G-CSF treatment mitigates AD neuropathology in the aged APP/PS1 mice (Guo et al., 2020), we aimed to explore the underlying mechanism of SCF+G-CSF treatment in this model at an advanced stage of disease using transcriptomic analysis. To accomplish this, 17–18-month-old APP/PS1 mice received subcutaneous (s.c.) injections of SCF+G-CSF or the vehicle solution for 12 consecutive days. One day after the final injection, RNA was extracted from the frontal lobes and used for gene chip analysis (Figure 1).

**FIGURE 1:**
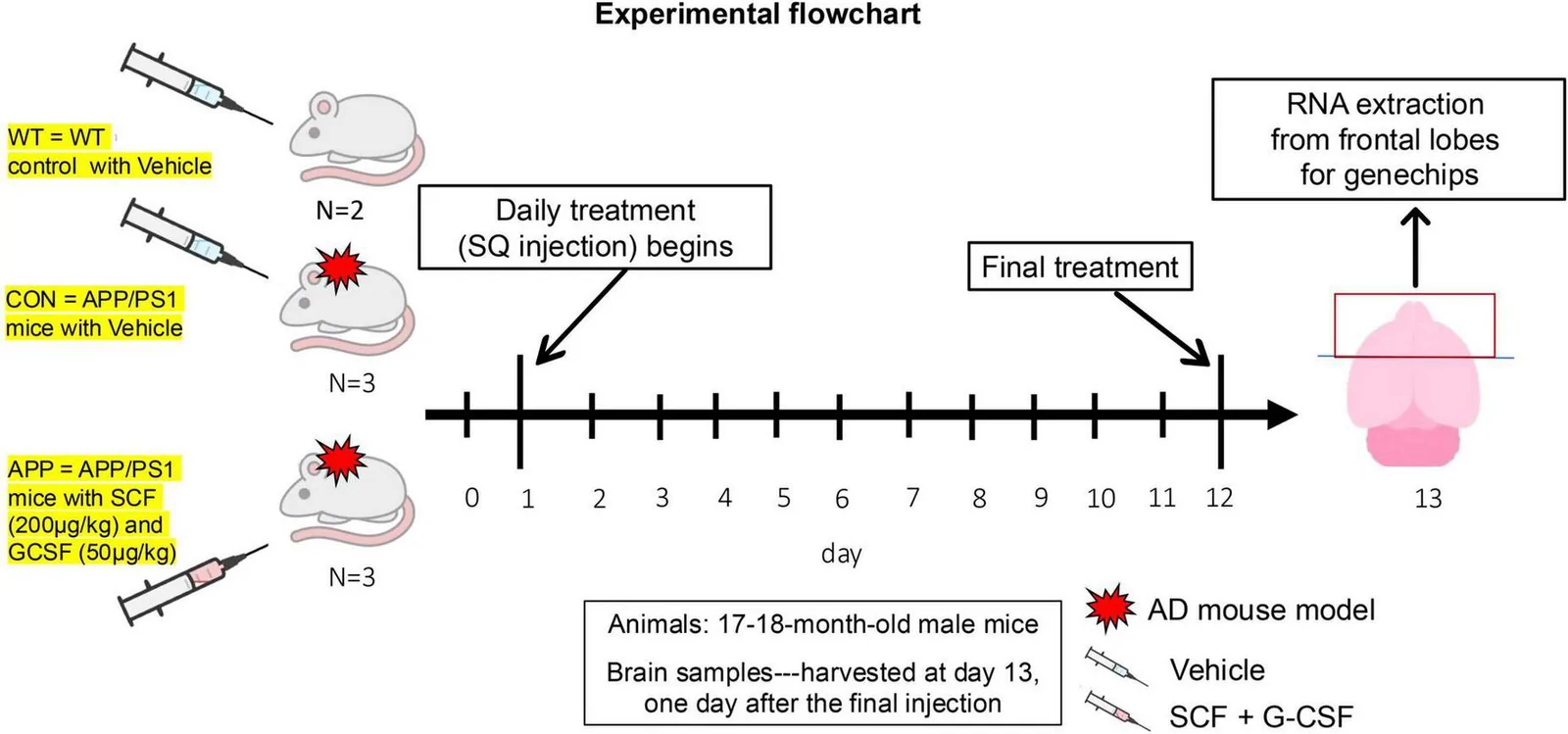
Schematic of experimental design. Male APP/PS1 mice (17–18 months old) were randomly divided into two groups (*n* = 3/group): one received SCF+G-CSF treatment and the other received vehicle injections. Age-matched WT mice (*n* = 2) receiving vehicle injections served as controls. All mice received daily subcutaneous (SQ) injections of SCF+G-CSF or vehicle for 12 consecutive days. On day 13, mice were euthanized, and RNA was extracted from frontal cortices for transcriptomic analysis using gene chip microarrays.

The APP/PS1 mouse model is widely used to study AD. This doubly transgenic model carries mutations in both the human APP and PS1 genes, leading to age-dependent amyloid plaque formation and gliosis (Janota et al., 2015; Xue et al., 2022). In this model, plaque deposition and gliosis typically begin early in life and show cognitive decline and neuroinflammatory changes (Denver et al., 2018; Huang et al., 2016; Janota et al., 2015; Xue et al., 2022).

For the present study, we used aged APP/PS1 mice to assess the transcriptional changes induced by SCF+G-CSF at a more advanced stage of AD-like pathology. Progressive, age-dependent increases in amyloid deposition, neuroinflammation, and immune dysfunction have been well documented in APP/PS1 mice (Abdelmoaty et al., 2023; Xue et al., 2022). For example, it has been reported that 12- and 20-month-old APP/PS1 mice exhibited approximately 27- and 67-fold increases, respectively, in cortical pan-Aβ burden compared with young mice, accompanied by exacerbated neuroinflammatory changes (Abdelmoaty et al., 2023). Thus, compared with younger APP/PS1 cohorts, aged mice exhibit substantially greater amyloid burden and neuroinflammatory pathology, providing a relevant model for exploring the molecular mechanisms and biological pathways associated with SCF+G-CSF treatment in the setting of advanced AD-like pathology. Consistent with this rationale, the therapeutic potential of SCF+G-CSF has also been investigated in aged APP/PS1 mice with more advanced pathology (Guo et al., 2020). Moreover, mice approximately 18–24 months of age are generally considered aged and are widely used to model late adulthood and aging in humans.

### 3.1 SCF+G-CSF treatment may counter Alzheimer’s disease-associated transcriptional changes through the regulation of key genes

Gene expression analysis using ThermoFisher Scientific’s TAC platform revealed that SCF+G-CSF treatment induces differential expression in a small set of genes (Figure 2A). A list of differentially expressed genes (DEGs) in SCF+G-CSF-treated APP/PS1 mice (APP) compared to untreated APP/PS1 mice (CON) is presented in Table 1. Out of the 45,037 genes identified, only 27 genes were significantly differentially expressed and met the two-fold expression change threshold. Among these 27 DEGs, 19 were downregulated, and 8 were upregulated following SCF+G-CSF treatment (Figures 2A,B). A heatmap representing hierarchical clustering of the dataset is shown in Figure 3, with red indicating upregulated genes and blue indicating downregulated genes.

**FIGURE 2:**
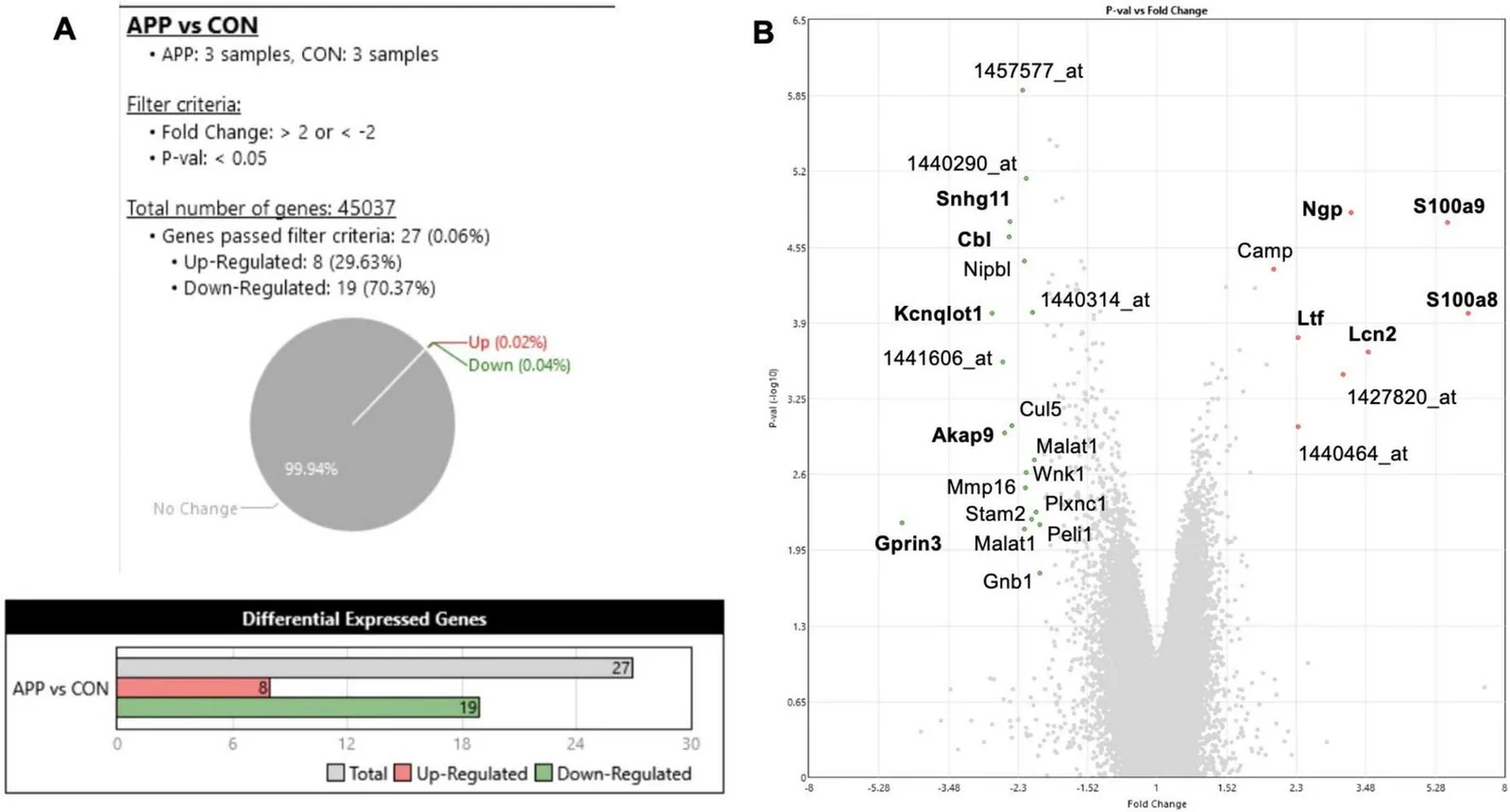
Summary and volcano plot of gene chip analysis using Transcriptome Analysis Console (TAC) in aged APP/PS1 mice treated with or without SCF+G-CSF. **(A)** SCF+G-CSF treatment significantly altered the expression of 27 genes, with over 70% being downregulated. **(B)** The top 5 upregulated and downregulated differentially expressed genes (DEGs) are highlighted in bold. Genes labeled with numerical probe set IDs lack known gene names or symbols. APP, APP/PS1 mice treated with SCF+G-CSF. CON, APP/PS1 control mice receiving vehicle injections.

**TABLE 1:** SCF+G-CSF-induced DEGs in the brains of aged APP/PS1 mice.

ID count: 27	APP Avg (log2)	CON Avg (log2)	Fold Chan…	*P*-val	FDR *P*-val	Gene symbol	Description
1419394_s_at	11.04	8.35	6.43	0.0001	0.1603	S100a8	S100 calcium binding pro…
1448756_at	10.38	7.88	5.66	1.72E-05	0.0862	S100a9	S100 calcium binding pro…
1427747_a_at	7.32	5.5	3.54	0.0002	0.2248	Lcn2	Lipocalin 2
1418722_at	8.88	7.21	3.19	1.43E-05	0.0862	Ngp	Neutrophilic granule prot…
1427820_at	9.79	8.18	3.04	0.0003	0.2886		
1450009_at	7.96	6.74	2.32	0.0002	0.199	Ltf	Lactotransferrin
1440454_at	7.34	6.13	2.32	0.001	0.4309		
1419691_at	7.79	6.78	2.01	4.32E-05	0.1338	Camp	Cathelicidin antimicrobial…
1454696_at	9.93	10.95	−2.02	0.0176	0.6726	Gnb1	Guanine nucleotide bindi…
1431060_at	4.68	5.7	−2.02	0.0068	0.6726	Peli1	Pellino 1
145O9O6_at	7.18	8.23	−2.06	0.0053	0.6726	Plxc1	Plexin C1
1438403_s_at	11.05	12.11	−2.09	0.0019	0.5414	Malat1	Metastasis associated lun…
1440314_at	6.2	7.28	−2.1	0.0001	0.1603		
1440034_at	5.59	6.67	−2.12	0.0061	0.6726	Stam2	Signal transducing adapt…
1440290_at	6.8	7.93	−2.18	7.16E-06	0.0806		
1436746_at	9.25	10.38	−2.19	0.0024	0.6066	Wnk1	WNK lysine deficient prot…
1440161_at	6.6	7.74	−2.2	0.0033	0.6726	Mmp16	Matrix metallopeptidase 16
1430309_at	8.94	10.08	−2.21	3.71E-05	0.1338	Nipbl	Nipped-B homolog (Dros…
1418188_a_at	10.45	11.59	−2.21	0.0074	0.6726	Malat1	Metastasis associated lun…
1457577_at	5.44	6.6	−2.23	1.26E-06	0.0569		
1452722_a_at	7.29	8.55	−2.38	0.001	0.4309	Cul5	Cullin 5
1437311_at	10.89	12.16	−2.41	1.69E-05	0.0862	Snhg11	Small nucleolar RNA host…
1455886_at	8.73	10.01	−2.42	2.29E-05	0.1029	Cbl	Casitas B-lineage lympho…
1437082_at	7.28	8.6	−2.5	0.0011	0.4695	Akap9	A kinase (PRKA) anchor p…
1441606_at	6	7.33	−2.52	0.0003	0.2556		
1442029_at	5.34	6.76	−2.68	0.0001	0.1603	Kcnq1ot1	KCNQ1 overlapping trans…
1439566_at	6.06	8.26	−4.6	0.0065	0.6726	Gprin3	GPRIN family member 3

Red-highlighted fold changes represent SCF+G-CSF-upregulated differentially expressed genes (DEGs), whereas green-highlighted fold changes represent SCF+G-CSF-downregulated DEGs compared with APP/PS1 control mice. Color gradients indicate the relative magnitude of the fold changes within groups. APP and CON averages represent the microarray signal intensities for each gene. Data were generated using the Transcriptome Analysis Console (TAC). APP, APP/PS1 mice treated with SCF+G-CSF; CON, APP/PS1 vehicle control mice.

**FIGURE 3:**
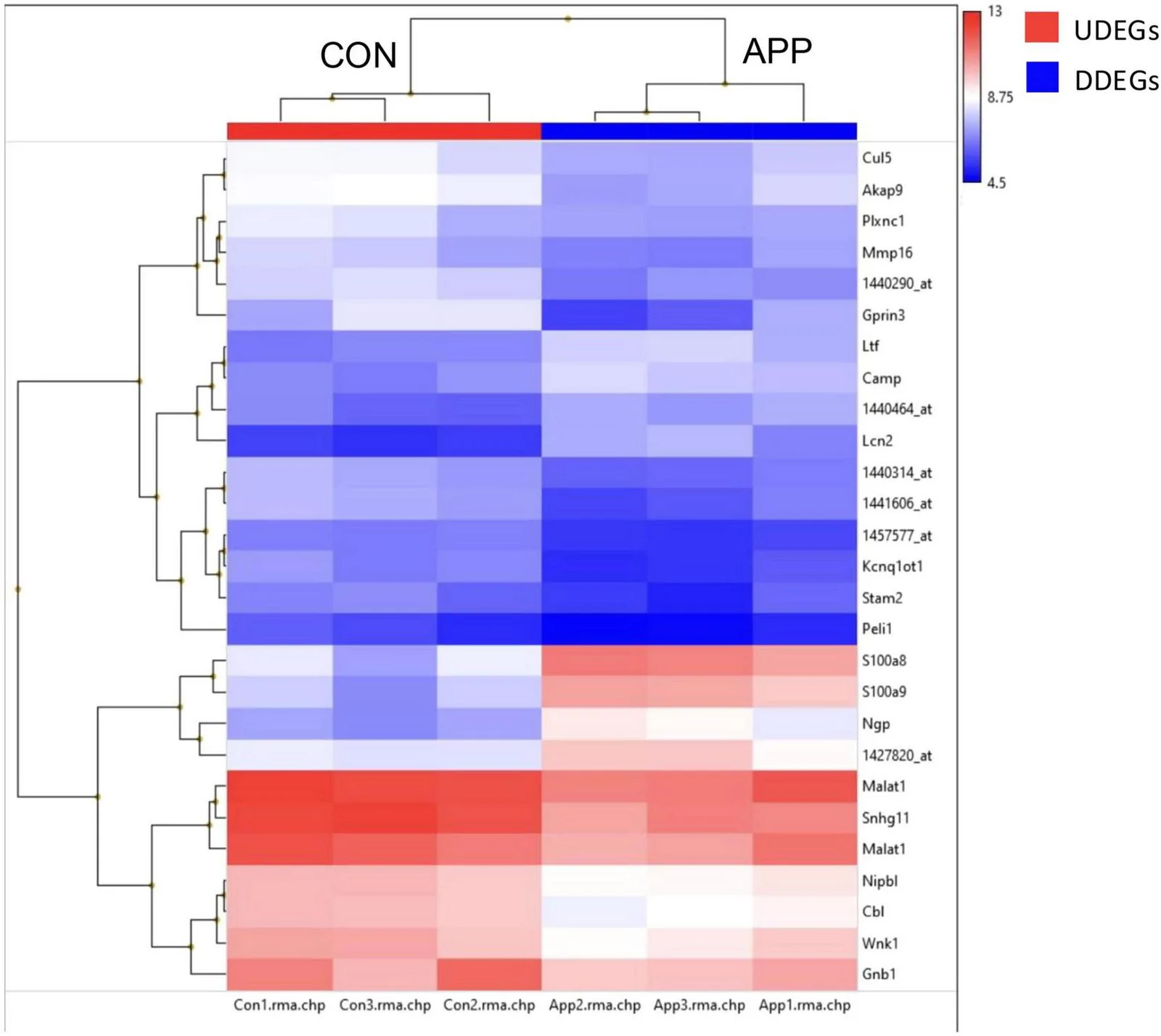
Heat map showing hierarchical clustering of differentially expressed genes (DEGs) in the brains of aged APP/PS1 mice treated with or without SCF+G-CSF. Normalized expression levels of DEGs are presented on a log-scale gradient, with darker colors indicating higher expression and lighter colors indicating lower expression. Red denotes upregulated DEGs (UDEGs), and blue indicates downregulated DEGs (DDEGs). The heat map was generated using the Transcriptome Analysis Console (TAC). APP, APP/PS1 mice treated with SCF+G-CSF. CON, APP/PS1 control mice receiving vehicle injections.

Analysis using ShinyGO v0.77 revealed that significantly more coding genes were differentially expressed in the APP/PS1-SCF+G-CSF (APP) group compared to APP/PS1 controls (CON) than would be expected by chance (*p* = 0.0025, Supplementary Figure S1A). Coding genes provide the genetic instructions that are transcribed into mRNA and then translated into proteins. Our dataset also showed that a few genes related to long non-coding RNAs (IncRNAs) were significantly altered following SCF+G-CSF treatment (Supplementary Figure S1A). IncRNAs are increasingly being recognized for their role in regulating transcription, chromatin organization, RNA processing, and post-translational gene regulation (Statello et al., 2021).

These findings suggest that SCF+G-CSF treatment may preferentially modulate the expression of coding genes while also influencing regulator non-coding transcripts, thereby influencing both transcriptional and translational processes. Supplementary Figure S1B illustrates a chromosomal map of DEGs identified from TAC analysis, further confirming that SCF+G-CSF treatment predominantly drives gene downregulation. The base pair localization of each gene on its respective chromosome is provided in Supplementary Table S1. Together, these data indicate that most genes affected by SCF+G-CSF treatment are suppressed coding genes in aged APP/PS1 mice. Thus, even modest transcriptional modulation may help reduce AD-related neuropathology.

### 3.2 Functions and implications for SCF+G-CSF-upregulated differentially expressed genes

All SCF+G-CSF-upregulated DEGs are shown in Figure 2B and Table 1. The functions and implications of the 6 known DEGs following SCF+G-CSF treatment are inferred from transcriptomic analysis of gene chip data and interpreted in the context of previously published studies.

*S100a8 and S100a9.* The two most highly upregulated DEGs following SCF+G-CSF treatment were *S100a8* and *S100a9*, which increased by 6.43- and 5.66-fold, respectively (Table 1). To validate these findings, qRT-PCR was performed using an independent cohort of aged APP/PS1 mice because the original brain samples used for the gene-chip analysis were no longer available. Consistent with the gene-chip results, SCF+G-CSF treatment significantly increased *S100a8* and *S100a9* mRNA expression (Supplementary Figure S2), supporting the reproducibility of the treatment-induced upregulation of these genes.

*S100a8* and *S100a9* genes encode cytoplasmic calcium-binding proteins that can form either homodimers or heterodimers with each other as well as participate in diverse inflammatory and immune regulatory processes (Markowitz and Carson, 2013; Pruenster et al., 2016). Members of the S100 protein family have been implicated in amyloid plaque aggregation and neuroinflammatory signaling pathways associated with AD neuropathology (Cristovao and Gomes, 2019; Hagmeyer et al., 2019).

Although S100A8/A9 proteins are often associated with proinflammatory states and have been implicated in amyloid-related pathology, their biological functions are complex and may not be exclusively harmful (Cristovao and Gomes, 2019; Hagmeyer et al., 2019; Ma et al., 2017). Of note, localization of S100 proteins to amyloid plaques does not necessarily indicate a direct role in amyloidogenesis, as the functions of this protein family are far more diverse. S100A8 and S100A9 proteins are critically involved in the recruitment of arachidonic acid from the cell membrane, have been linked to NADPH oxidase (NOX) associated inflammatory responses, and regulation of oxidative signaling pathways (Lim et al., 2009). Other functions include innate immune system signaling, antimicrobial responses, leukocyte recruitment, and even cytoskeletal regulation (Ma et al., 2017; Pruenster et al., 2016; Wolf et al., 2023).

Accumulating evidence suggests that upregulation of S100A9 promotes the differentiation of promyelocytes into myelocytes or granulocytes, facilitating myeloid maturation (Markowitz and Carson, 2013). This process is critical for the CNS response to amyloid deposition, as bone marrow- or myeloid-derived cells, such as macrophages, can adopt microglia-like phenotypes and act as CNS phagocytes, which are key contributors to amyloid clearance (Malm et al., 2005; Yan et al., 2022). Additionally, S100A8 and S100A9 have been found to sequester metal ions like zinc to limit bacterial growth (Lim et al., 2009) and interact with microtubules to promote phagocyte migration (Hagmeyer et al., 2019; Wolf et al., 2023).

Another study reported that loss of S100A8/S100A9 led to dysregulation of phagocyte migration, cytoskeletal rearrangement, and cell adhesion (Wolf et al., 2023). These functions resemble microtubule-related processes involved in neurofibrillary tangle (NFT) formation, which are critical for intracellular trafficking. Collectively, these functions suggest that the SCF+G-CSF- induced upregulation of *S100a8* and *S100a9* may be associated with enhanced innate immune modulation, myeloid cell activation, and phagocyte-associated amyloid clearance. These transcriptional changes provide mechanistic insight into the immunomodulatory effects of SCF+G-CSF and identify candidate pathways for future investigation.

Our transcriptomic analysis identified robust upregulation of *S100a8* and *S100a9* following SCF+G-CSF treatment, suggesting enhanced immunomodulatory activity associated with myeloid cell responses. We further propose that the enrichment of the IL-17 signaling pathway may be associated with the increased expression of *S100a8* and *S100a9*, identifying a potential immunomodulatory pathway that warrants further investigation (Supplementary Figure S3). However, the present study does not determine whether these transcriptional changes influence peripheral immune cell trafficking or recruitment into the brain. Future studies incorporating analyses of peripheral immune cell populations, chemokine and chemokine receptor expression, and complementary blood and brain assessments (e.g., flow cytometry and targeted molecular validation) will be important to define the cellular mechanisms underlying these transcriptomic changes. This proposed model is consistent with previous studies demonstrating that microglia are activated by and secrete inflammatory cytokines, including IL-6 and TNFα, through TLR4-dependent signaling (Ma et al., 2017).

The diverse biological functions of S100A8 and S100A9 reported in the literature, together with our transcriptomic findings, suggest that these proteins are more likely involved in immune regulation and amyloid-associated responses than in amyloid production (Cristovao and Gomes, 2019; Hagmeyer et al., 2019; Pruenster et al., 2016). We propose that the SCF+G-CSF-induced upregulation of *S100a8* and *S100a9*, together with other coordinately upregulated genes (Figure 4), represents a candidate immunomodulatory transcriptional program that may influence microglial and myeloid cell responses in the aged APP/PS1 brain. Based on previous studies, this transcriptional program may be relevant to immune cell activation, amyloid-associated immune responses, and Aβ clearance (Cristovao and Gomes, 2019; Malm et al., 2005; Yan et al., 2022). However, the specific cellular mechanisms and functional consequences of these transcriptional changes remain to be determined through future mechanistic studies.

**FIGURE 4:**
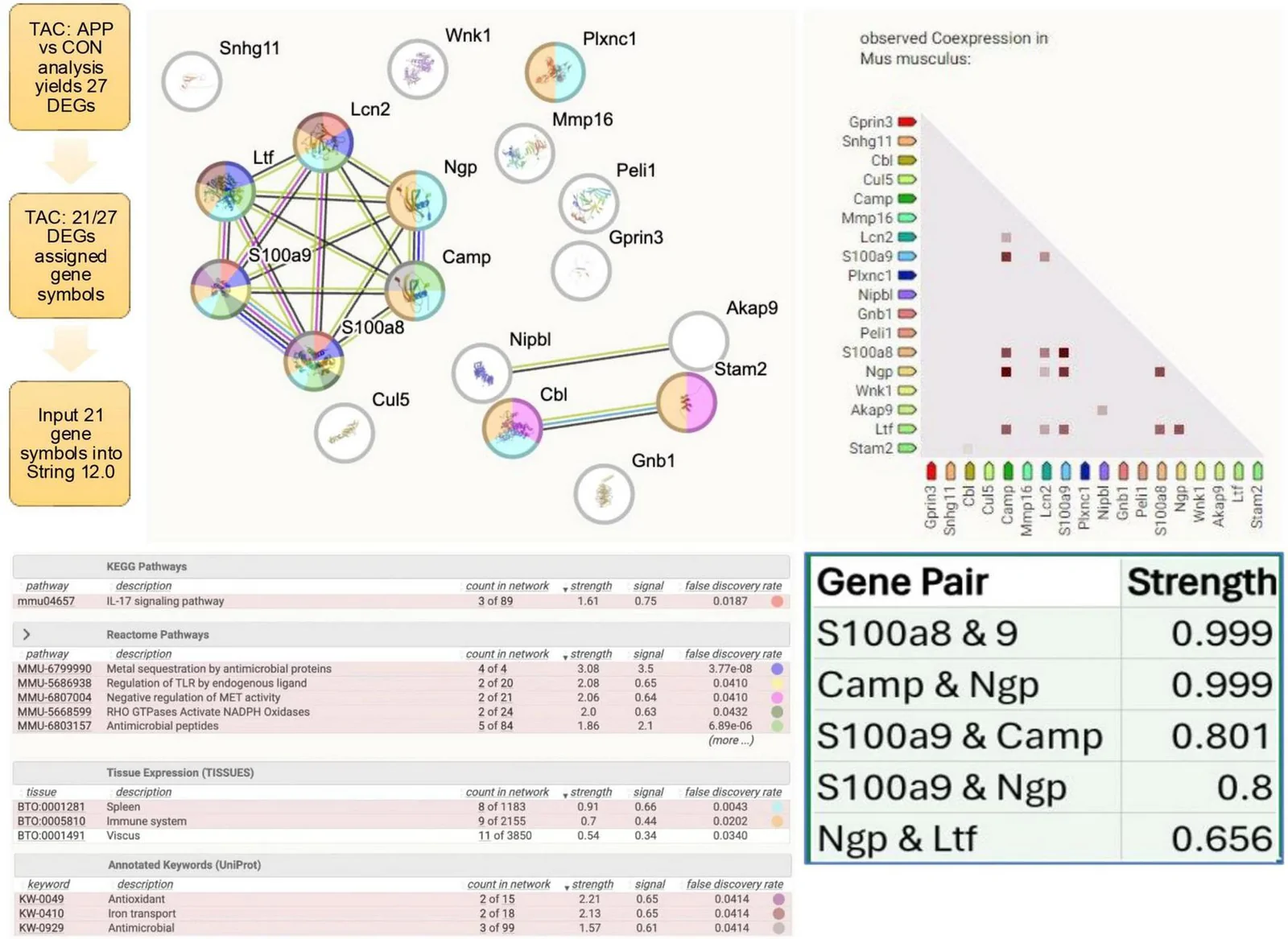
STRING 12.0 analysis of SCF+G-CSF-induced differentially expressed genes (DEGs) in the brains of aged APP/PS1 mice. Transcriptome Analysis Console (TAC) analysis identifies 27 DEGs in response to SCF+G-CSF treatment, of which 21 have annotated gene names. Among these 21 DEGs, 18 genes are identified in the STRING 12.0 interaction analysis. STRING analysis identifies a six-member interacting and co-expressed gene cluster previously associated with spleen and immune tissues, which is also present in the brains of APP/PS1 mice. Functional annotation indicates that these genes are associated with IL-17 signaling, metal sequestration, regulation of Toll-like receptor (TLR) signaling by endogenous ligands, negative regulation of MET activity, RHO GTPase-mediated activation of NADPH oxidases, and antimicrobial peptide pathways.

*Lcn2. Lcn2* was upregulated 3.54-fold following SCF+G-CSF treatment in aged APP/PS1 mice (Table 1). Also known as neutrophil gelatinase-associated lipocalin (NGAL), Lcn2 belongs to the lipocalin protein family, whose members regulate diverse biological processes, including iron transport, immune regulation, and cell proliferation and migration (Jaberi et al., 2021; Yang et al., 2023). Lcn2 is also upregulated in various cancers (Adler et al., 2023; Yang et al., 2023) and was originally identified as a component of neutrophil granules, where it facilitates matrix metalloproteinase 9 activity and contributes to extracellular matrix (ECM) remodeling (Jaberi et al., 2021).

Although some studies suggest that Lcn2 exerts primarily harmful effects, the literature remains divided (Ferreira et al., 2015; Kang et al., 2018). Lcn2 is highly expressed in the brains of mild cognitive impairment, AD, and vascular dementia patients (Dekens et al., 2017; Llorens et al., 2020). One study reported that Lcn2 levels were elevated in the hippocampus and amygdala of AD human tissue samples (Dekens et al., 2017). Another study suggested that Lcn2 may exert neuroprotective effects, including regulating protein concentrations of IL-6, IL-1β, and TNFα, in response to systemic inflammation, as Lcn2 knockout (KO) mice exhibited elevated peripheral pro-inflammatory cytokines and impaired behavioral phenotypes compared with WT mice (Kang et al., 2018).

Additional studies have demonstrated that *Lcn2* KO mice exhibit increased susceptibility to *E. Coli* infection and reduced bacteriostatic activity (Berger et al., 2006; Goetz et al., 2002). During bacterial infection, lipopolysaccharide (LPS) stimulation of macrophage surface receptors induces apoptosis and cell shrinking, and under these conditions, Lcn2 functions as an acute-phase reactant (Ferreira et al., 2015; Flo et al., 2004; Jaberi et al., 2021). Lcn2 also promotes iron sequestration to limit bacterial access to metal ions (Flo et al., 2004), a process that may be relevant to AD pathogenesis given the contribution of metal dyshomeostasis to neurodegeneration (Chen et al., 2023; Flo et al., 2004; Liu et al., 2022).

These diverse functions suggest that Lcn2 is an important regulator of innate immune responses. Lcn2 has also been implicated in immune cell recruitment and neuroinflammatory signaling within the brain (Adler et al., 2023). Consistent with these observations, our transcriptomic analysis identified both *Lcn2* upregulation and enrichment of the IL-17 signaling pathway following SCF+G-CSF treatment (Figure 4 and Supplementary Figure S3), suggesting a potential immunomodulatory pathway that warrants further mechanistic investigation.

When evaluating a gene such as *Lcn2*, both its potentially detrimental and beneficial functions should be considered. Pro-inflammatory signaling is an essential component of host immune responses to diverse challenges, including pathogens and Aβ accumulation. Our transcriptomic analysis identified *Lcn2* as part of an SCF+G-CSF-upregulated gene cluster, suggesting a potential role in treatment-associated immunomodulation in the aged APP/PS1 brain (Figure 4). Similar to S100A8 and S100A9, Lcn2 participates in antibacterial defense as a component of neutrophil granules, supporting the concept that inflammatory responses are not inherently detrimental but can also contribute to immune regulation and surveillance. Collectively, these findings, together with previous studies, suggest that controlled neuroinflammatory responses may participate in the brain’s response to AD-related pathology. However, the precise functional role of Lcn2 and the broader SCF+G-CSF-induced transcriptional program remain to be determined through future mechanistic studies.

*Ngp. Ngp* was upregulated 3.19-fold in the brains of aged APP/PS1 mice following SCF+G-CSF treatment (Table 1). *Ngp* encodes the neutrophil granule protein. Ngp was originally identified in murine bone marrow, where it is enriched in immature neutrophil-lineage cells and stored within the cytoplasm of specific cell types (Moscinski and Hill, 1995; Othman et al., 2022). Granule-associated proteins such as Ngp have been implicated in regulating immune responses, antimicrobial defense, and phagocytic functions (Othman et al., 2022).

In the context of neuroinflammation, NETosis, the formation of neutrophil extracellular traps (NETs), is triggered by inflammatory cytokines and depends on NADPH oxidase signaling. During this process, neutrophils release extracellular chromatin, histones, and granule proteins that contribute to pathogen immobilization and clearance (Mutua and Gershwin, 2021; Vorobjeva and Chernyak, 2020; Zhao et al., 2021). NETosis is increasingly recognized as part of a broader spectrum of inflammatory cell death programs implicated in chronic disease and tissue injury, rather than solely in antimicrobial defense (Zhao et al., 2021). In AD, NET formation has been associated with neuroinflammatory and vascular pathology, suggesting a potential role in disease-associated immune dysregulation (Pietronigro et al., 2017).

The SCF+G-CSF-induced upregulation of *Ngp* observed in the present study, together with previous reports of its immunological function, suggests that Ngp may participate in treatment-associated immune responses in the aged APP/PS1 mouse brain. Because Ngp is a granule-associated protein, its increased expression may also be associated with transcriptional programs related to innate immune activation, including pathways linked to neutrophil biology and NETosis. However, the cellular source of Ngp and its contribution to the immunomodulatory effects of SCF+G-CSF require further investigation.

*Ltf. Ltf* was upregulated 2.32-fold following SCF+G-CSF treatment in the brains of aged APP/PS1 mice (Table 1). Also known as lactotransferrin, Ltf participates in iron sequestration, immune modulation, antimicrobial, antiviral, antioxidant, anti-inflammatory, and anticancer activities, as well as broader regulatory functions in inflammation and host defense (Hao et al., 2019). Within the CNS, Ltf is increasingly being recognized as a multifunctional mediator involved in neuroimmune regulation and disease-associated signaling (Li and Guo, 2021). Consistent with this role, increased Ltf expression has been reported in brain regions enriched with neurofibrillary tangles and senile plaques in AD patients (Li and Guo, 2021). Previous studies have also suggested that Ltf may be produced by microglia and contribute to neuroprotective functions (Li and Guo, 2021). These findings suggest a potential role for Ltf in neuroimmune responses associated with amyloid pathology in AD.

Additionally, Ltf transport across the blood-brain barrier (BBB) is thought to be modulated under inflammatory conditions and has been linked to iron homeostasis and neurovascular signaling (Khan et al., 2020), suggesting that Ltf may influence BBB function and inflammatory signaling in the CNS. Ltf protein is predominantly expressed by activated microglia and infiltrating monocytes/macrophages (Li and Guo, 2021), suggesting a potential role in neuroimmune crosstalk between resident and peripheral innate immune cells. Consistent with these observations, our recently published scRNA-seq analysis of brain CD11b^+^ cells from aged APP/PS1 mice, comprising resident microglia and infiltrating monocytes/macrophages, also identified *Ltf* as an upregulated gene following SCF+G-CSF treatment (Gardner et al., 2024), providing independent support for the SCF+G-CSF-induced upregulation of *Ltf* observed in the present study.

Another study investigating the effects of intranasal Ltf administration in APP/PS1 mice reported that Ltf treatment reduced Aβ plaque accumulation, attenuated synapse loss in the cortex and hippocampus, and improved spatial learning (Guo et al., 2017). The reduction in Aβ plaque burden was associated with enhanced non-amyloidogenic, α-secretase-dependent processing of amyloid precursor protein (APP), thereby limiting Aβ production (Guo et al., 2017).

Collectively, these findings suggest that Ltf has context-dependent functions and may participate in both immune regulation and neuroprotective signaling. The SCF+G-CSF-induced upregulation of *Ltf* observed in the present study further supports its potential involvement in treatment-associated immunomodulatory responses in the aged APP/PS1 mouse brain. However, the functional significance of *Ltf* upregulation following SCF+G-CSF treatment remains to be established.

*Camp*. *Camp* was upregulated 2.01-fold following SCF+G-CSF treatment in the brains of aged APP/PS1 mice (Table 1). It is one of six immune-related genes comprising the SCF+G-CSF-upregulated transcriptional cluster (Figure 4), suggesting a potential role in treatment-associated immunomodulation. *Camp* encodes cathelicidin antimicrobial peptide (CAMP), also known as cathelicidin-related antimicrobial peptide in mice. In an LPS-induced neuroinflammation model, cathelicidin-related antimicrobial peptide expression was increased in glial and neighboring neural cells, and administration of the peptide reduced inflammatory activation of microglia and astrocytes (Bhusal et al., 2022). Based on these findings, we postulate that the SCF+G-CSF-induced upregulation of *Camp* may contribute to treatment-associated modulation of inflammatory signaling in the brains of aged APP/PS1 mice.

Given Camp’s well-established antimicrobial properties, it is not surprising that it has increasingly been investigated for its role in wound healing. Wang and colleagues reported that Camp promotes angiogenesis-related activities and wound healing through activation of MAP/ERK-associated signaling pathways (Wang et al., 2022). These reparative properties may also be relevant to the biological effects associated with SCF+G-CSF treatment. Camp has also been found to be directly induced by vitamin D receptor activation in myeloid cells (Gombart et al., 2005), and a subsequent study further demonstrated vitamin D-induced Camp expression in mouse models (Lowry et al., 2020).

Beyond genetic and aging-related factors, viral and bacterial infection, as well as alterations in the gut microbiome, have been implicated in AD pathogenesis through their ability to modulate neuroinflammatory signaling. An epidemiological study reported that viral infections, including herpes simplex virus (HSV), are associated with an increased risk of AD (Steel and Eslick, 2015). Infection may activate the innate immune system and promote neuroinflammatory responses implicated in AD pathology (Rajesh and Kanneganti, 2022). Under these conditions, activated glial cells may further amplify inflammatory signaling cascades (Orihuela et al., 2016; Rajesh and Kanneganti, 2022).

Although the mechanisms linking specific pathogens to amyloid pathology remain incompletely understood, these findings collectively support an important role for neuroinflammatory processes in AD. In this context, the SCF+G-CSF-induced upregulation of *Camp* may reflect a treatment-associated immunomodulatory response in the aged APP/PS1 brain. However, the functional significance of increased *Camp* expression following SCF+G-CSF treatment requires further investigation.

### 3.3 Functions and implications for SCF+G-CSF-downregulated differentially expressed genes

The five most highly downregulated differentially expressed genes (DEGs) following SCF+G-CSF treatment are shown in Figure 2B and Table 1. Their potential biological functions and implications are inferred from transcriptomic findings and interpreted in the context of previously published studies.

*Gprin3. Gprin3* was the most highly downregulated DEG in our dataset, exhibiting a 4.60-fold decrease following SCF+G-CSF treatment in the brains of aged APP/PS1 mice (Table 1). *Gprin3* encodes G-protein-regulated inducer of neurite outgrowth 3 (GPRIN3) (Karadurmus et al., 2019; Mototani et al., 2018). Members of the GPRIN family are highly expressed in inhibitory medium spiny neurons of the striatum, where they modulate dopamine D2 receptor signaling and regulate neuronal morphology and excitability (Karadurmus et al., 2019). Mechanistically, GPRIN proteins function downstream of G protein-coupled receptors and are linked to β-arrestin-2/Akt pathways that regulate neurite growth and striatum-dependent behaviors (Mototani et al., 2018).

Genetic deletion of *Gprin3* has been reported to increase dendritic arborization while reducing neuronal excitability (Karadurmus et al., 2019). Notably, our previous study demonstrated that SCF+G-CSF treatment restored dendritic loss in the cortex and hippocampus of aged APP/PS1 mice (Guo et al., 2020). Together, these findings suggest that SCF+G-CSF-induced downregulation of *Gprin3* may be associated with dendritic remodeling or repair in the setting of AD-related pathology. However, whether *Gprin3* directly contributes to these structural changes requires further investigation.

*Kcnq1ot1. Kcnq1ot1* was downregulated 2.68-fold following SCF+G-CSF treatment in aged APP/PS1 mice (Table 1). *Kcnq1ot1* encodes a long non-coding RNA (lncRNA) rather than a protein and has been implicated in cancer progression through regulation of cell growth and proliferation by modulating the transcription of protein-coding genes (Rapetti-Mauss et al., 2020; Statello et al., 2021). Consequently, *Kcnq1ot1* has been associated with diverse biological processes, including cell cycle regulation, migration, invasion, angiogenesis, and immune responses in cancer (Cagle et al., 2021).

Located on the antisense strand of the *KCNQ1* (potassium voltage-gated channel subfamily Q member 1) protein-coding gene (Cagle et al., 2021), *Kcnq1ot1* has been implicated in several solid tumors, including breast, lung, and gastrointestinal cancers (Cagle et al., 2021), as well as hematologic malignancies, particularly acute promyelocytic leukemia (APL) (Tang et al., 2021). It has also been investigated as a potential biomarker for neurological and developmental disorders, including AD (Ghiam et al., 2022; Jaberi et al., 2023).

Ion channels such as KCNQ1 are increasingly recognized for their roles in cancer biology through signaling pathways, including Wnt/β-catenin, MEK/ERK, and MAPK (Cagle et al., 2021; Huang et al., 2022; Llorens et al., 2020; Tang et al., 2021). Although APL is characterized by uncontrolled proliferation of immature granulocytes in the bone marrow (Fukuchi et al., 1998), the biological significance of *Kcnq1ot1* downregulation following SCF+G-CSF treatment in the aged APP/PS1 mouse brain remains unclear. Nevertheless, these findings raise the possibility that *Kcnq1ot1* participates in molecular pathways shared between cancer biology and neurodegeneration, warranting further investigation.

*Akap9. Akap9* is downregulated 2.50-fold following SCF+G-CSF treatment in aged APP/PS1 mice (Table 1). *Akap9* encodes A-kinase anchoring protein 9 (AKAP9), which participates in T-cell-antigen-presenting cell interactions, receptor recycling, and regulation of microtubule dynamics (Herter et al., 2015). Deletion of *Akap9* in CD4^+^ and CD8^+^ T cells has been reported not to affect T-cell priming, expansion, or migration to inflamed tissues, but to reduce T-cell reactivation and retention at these sites (Herter et al., 2015).

In AD and neurodegenerative disorders, *Akap9* has also been implicated in tau regulation. Rare *Akap9* variants have been associated with an increased risk of AD in African Americans, potentially through altered tau phosphorylation, leading to the investigation of *Akap9* as a candidate biomarker in this population (Ikezu et al., 2018). Specifically, lymphoblastoid cell lines derived from African Americans with AD carrying *Akap9* variants exhibited significantly altered tau phosphorylation independent of *APOE* genotype or amyloid precursor protein expression (Ikezu et al., 2018). These findings are particularly noteworthy given the underrepresentation of African Americans in AD research.

In 2022, You and colleagues reported that neuronal models carrying *Akap9* mutations exhibited altered post-translational modification of tau and changes in the tau protein interactome, further supporting a role for AKAP9 in tau-associated cellular processes (You et al., 2022).

Like *Kcnq1ot*, increased *Akap9* expression has been associated with acute myeloid leukemia (Tang et al., 2021; Wu et al., 2022) and has been linked to Wnt/β-catenin signaling, an important oncogenic pathway (Wu et al., 2022). In addition, our pathway enrichment analysis identified negative regulation of MET activity following SCF+G-CSF treatment (Figure 4 and Supplementary Figure S4). MET signaling is a well-established driver of tumor growth, metastasis, and pro-tumor metabolism across multiple cancer types (Davis and Waltz, 2024). Collectively, these findings raise the possibility that SCF+G-CSF modulates molecular pathways shared between cancer biology and neurodegeneration. The biological significance of *Akap9* downregulation and negative regulation of MET activity in the aged APP/PS1 brain remains to be elucidated.

*Cbl. Cbl* was downregulated 2.42-fold following SCF+G-CSF treatment in aged APP/PS1 mice (Table 1). *Cbl* encodes Casitas B-Lineage Lymphoma (CBL) protein, a member of the RING finger ubiquitin ligase family that functions as both a positive and negative regulator of signal transduction mediated by receptor and non-receptor tyrosine kinases (Kales et al., 2010).

Like *Kcnq1ot1*, *Cbl* has been implicated in hematologic malignancies, and dysregulation of CBL signaling has been associated with myeloid leukemogenesis in both mice and humans (Kales et al., 2010; Lyle et al., 2019). Consistent with these observations, our transcriptomic analysis identified *Cbl* downregulation following SCF+G-CSF treatment. In addition, TAC pathway analysis showed *Cbl* involvement in multiple signaling pathways, including IL-7, IL-2, IL-4, and EGFR1 (Figure 5). Reactome pathway analysis further identified *Cbl* in more than 40% of the enriched pathways, with approximately 50-fold pathway enrichment associated with CBL-regulated functions following SCF+G-CSF treatment (Figure 6).

**FIGURE 5:**
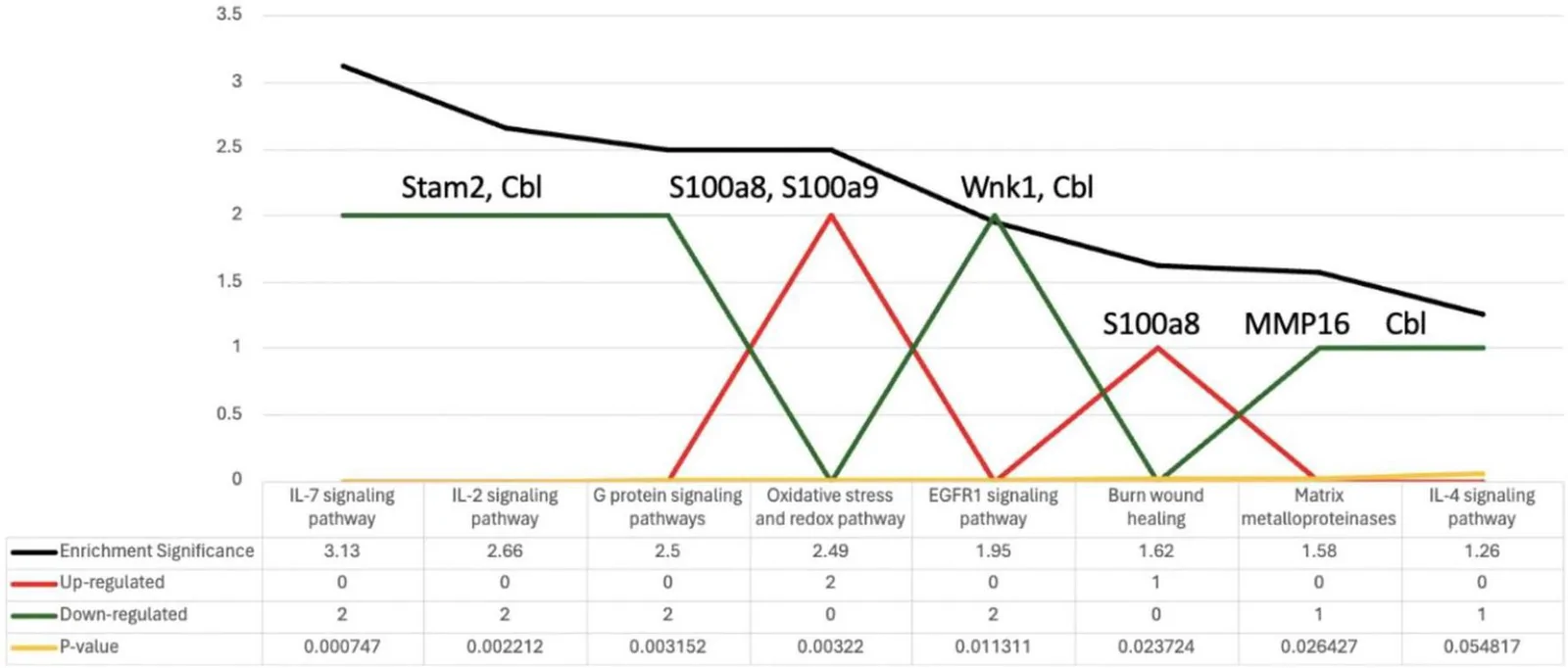
Transcriptome analysis console (TAC) pathway enrichment analysis of SCF+G-CSF-induced differentially expressed genes (DEGs) in the brains of aged APP/PS1 mice. SCF+G-CSF treatment enriches pathways associated with S100a8/a9-related oxidative stress and wound healing, while SCF+G-CSF-downregulated DEGs are associated with reduced enrichment of inflammatory pathways, EGFR1 signaling, and MMP16-related pathways.

**FIGURE 6:**
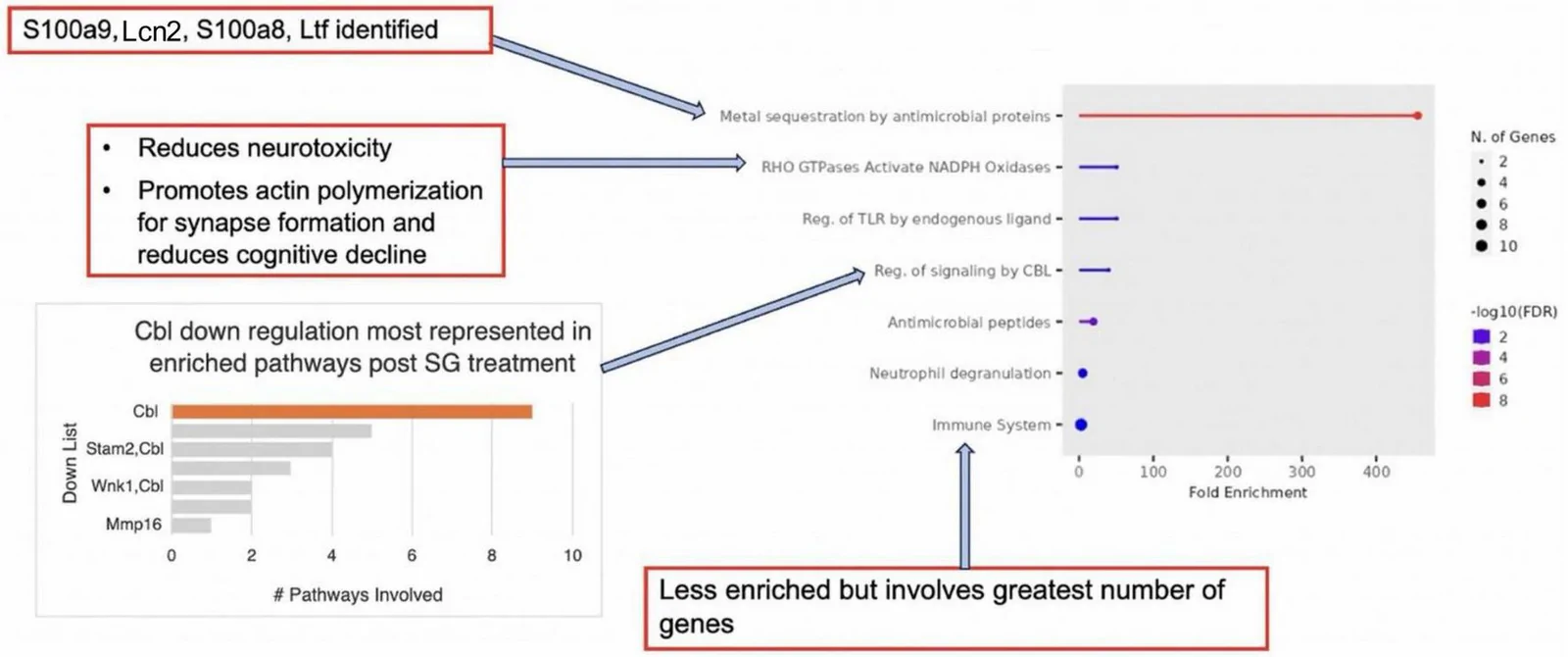
Reactome pathway analysis of SCF+G-CSF-induced differentially expressed genes (DEGs) in the brains of aged APP/PS1 mice. Reactome pathway enrichment analysis identifies pathways associated with metal sequestration, immune system regulation, synaptic function, and cancer-related processes following SCF+G-CSF treatment in aged APP/PS1 mice. The most enriched pathway is metal sequestration. Additional enriched pathways are associated with oxidative stress responses, synaptic remodeling, immune regulation, and the downregulation of *Cbl*, a cancer-associated gene represented in more than 40% of the enriched pathways.

Together, these findings suggest that *Cbl* may represent one component of the broader immunomodulatory transcriptional program induced by SCF+G-CSF. However, downregulation of *Cbl* alone does not provide direct evidence for altered cancer-associated signaling or reduced immunosuppression, and the functional significance of this observation remains to be established through future cell-specific and mechanistic studies.

Cancer and neurodegeneration share several biological processes, including chronic inflammation, DNA damage, oxidative stress, and metabolic dysfunction (Houck et al., 2018). Epidemiological studies have also reported an inverse association between AD and cancer risk. For example, a large cohort study involving more than 1,200 participants found that individuals with AD were less likely to develop cancer, whereas cancer survivors exhibited a lower risk of developing AD than individuals without cancer (Driver et al., 2012). In this context, the coordinated downregulation of *Cbl*, *Kcnq1ot1*, and *Akap9*, together with the negative regulation of MET activity identified by pathway enrichment analysis, raises the possibility that SCF+G-CSF influences molecular pathways shared between neurodegeneration and cancer biology. Although the biological significance of these transcriptional changes remains unclear, they provide a foundation for future studies examining the intersection of immune regulation, cancer-associated signaling, and AD pathology.

*Snhg11. Snhg11* was downregulated 2.41-fold following SCF+G-CSF treatment in aged APP/PS1 mice (Table 1). *Snhg11*, a long non-coding RNA (lncRNA), has been implicated in the activation of the Wnt/β-catenin signaling pathway in gastric cancer models (Wu et al., 2021) and in promoting tumor metastasis through interactions with hypoxia-inducible factor-1α (HIF-1α) (Xu et al., 2020). Elevated *Snhg11* expression has also been associated with poor cancer prognosis (Wu et al., 2021).

As discussed above, lncRNAs broadly regulate gene expression and molecular signaling networks across diverse biological systems (Statello et al., 2021). In a neurological context, *Snhg11* has also been implicated in regulating synaptic plasticity and neurogenesis in the dentate gyrus of Down syndrome-associated models (Sierra et al., 2024). Collectively, these findings highlight the diverse biological functions of *Snhg11* in both neurological disorders and cancer. The biological significance of *Snhg11* downregulation following SCF+G-CSF treatment in the aged APP/PS1 brain remains to be determined.

### 3.4 SCF+G-CSF-induced six-gene interaction network suggests coordinated reparative mechanisms in aged APP/PS1 mice

Our transcriptomic analysis suggests that SCF+G-CSF induces a selective transcriptional response associated with biological pathways relevant to AD pathology. STRING v12.0 network analysis identified a functionally connected six-gene cluster consisting of *S100a8*, *S100a9*, *Lcn2*, *Ngp*, *Ltf*, and *Camp* (Figure 4). Among the 27 DEGs identified following SCF+G-CSF treatment, six upregulated genes formed this highly interconnected network, whereas most remaining DEGs were downregulated (Figure 2). These findings suggest that SCF+G-CSF may preferentially modulate specific immune-related transcriptional programs rather than broadly altering gene expression.

The coordinated expression of this six-gene cluster suggests a potential role in treatment-associated immunomodulation. Among these genes, *S100a8* and *S100a9* have been implicated in amyloid localization, immune cell recruitment, and phagocyte-mediated amyloid clearance (Cristovao and Gomes, 2019; Hagmeyer et al., 2019; Pruenster et al., 2016). Notably, these two genes were the most highly upregulated DEGs following SCF+G-CSF treatment and exhibited an exceptionally strong co-expression correlation (*r* = 0.999) (Figure 4).

The biological functions and tissue distribution of genes within this network provide additional context for their potential relevance. STRING analysis associated this six-gene cluster with immune system- and spleen-related biological processes (Figure 4). As a major secondary lymphoid organ, the spleen plays a central role in coordinating peripheral immune responses. Previous work demonstrated that splenectomy reduced plaque-associated macrophages, increased cerebral amyloid burden, and worsened cognitive deficits in APP/PS1 mice, highlighting the importance of peripheral immune responses in AD pathogenesis (Yu et al., 2022). In the present study, upregulation of this six-gene cluster in the brains of aged APP/PS1 mice suggests that SCF+G-CSF may modulate coordinated immune responses involving both central and peripheral immune compartments. However, the mechanisms linking these transcriptional changes to functional immune responses remain to be determined.

### 3.5 SCF+G-CSF-induced enrichment of the IL-17 signaling pathway suggests an immunomodulatory mechanism in aged APP/PS1 mice

ShinyGO 0.77 analysis identified a 29.1-fold enrichment of the IL-17 signaling pathway following SCF+G-CSF treatment, with three genes (*S100a8, S100a9*, and *Lcn2)* from our dataset represented in this pathway (Supplementary Figure S3). STRING v12.0 analysis further identified functional associations among these genes (Figure 4). Based on these findings and a previous study (Ye et al., 2023), we hypothesize that SCF+G-CSF-induced enrichment of the IL-17 signaling pathway may be associated with the coordinated upregulation of *S100a8*, *S100a9*, and *Lcn2.* However, whether IL-17 directly regulates the expression of these genes remains to be elucidated.

The functional significance of IL-17 pathway enrichment in AD remains an area of active investigation, and the role of IL-17 in AD remains controversial. While some studies suggest that IL-17 promotes neuroinflammation, this inflammatory response may also be necessary for glial production of chemokines that recruit myeloid cells and T cells (Ye et al., 2023). Accordingly, anti-IL-17 (and anti-IL-23) antibodies have been proposed as potential therapeutic strategies for AD, and IL-17 ablation was shown to reduce CD8^+^ T-cell and myeloid cell infiltration into the brains of APP/PS1 mice (Ye et al., 2023). However, the functional consequences of T-cell infiltration in AD remain unclear. In APP/PS1 mice, CD8^+^ T cells are present in the brain and influence neuronal and synaptic gene expression; however, depletion of CD8^+^ T cells from the brain, spleen, and blood of APP/PS1 mice did not improve cognitive function or reduce amyloid plaque burden (Unger et al., 2020).

This raises an important question: If neuroinflammation is a hallmark of AD and IL-17 promotes this process, why does IL-17 ablation not attenuate AD pathology? Our study offers one possible explanation. Although IL-17 signaling may promote immune cell recruitment, it may also support beneficial immune responses involved in amyloid recognition and clearance. Our STRING analysis (Figure 4) identified *S100a8*, *S100a9*, and *Lcn2* as functionally associated genes within the IL-17 signaling pathway. Together with previous findings (Ye et al., 2023), we hypothesize IL-17 may modulate the coordinated expression of these genes, thereby creating a microenvironment conducive to immune cell infiltration into the brain and subsequent amyloid recognition and clearance. However, direct experimental studies will be required to determine whether IL-17 regulates these genes and to define their contribution to treatment-associated immunomodulation in AD.

Taken together, our ShinyGO (Supplementary Figure S3) and STRING (Supplementary Figure S4) analyses, together with our previous studies (Gardner et al., 2024; Guo et al., 2020), support the hypothesis that SCF+G-CSF treatment induces a coordinated immunomodulatory transcriptional program in the brains of aged APP/PS1 mice. We further hypothesize that IL-17 pathway enrichment may contribute to treatment-associated immune cell recruitment and amyloid clearance. An alternative interpretation, however, is that IL-17 signaling is not directly induced by SCF+G-CSF but instead represents an intrinsic component of AD pathology that is insufficiently suppressed by treatment. Future studies should investigate IL-17 pathway subtypes and their regulation using cell type-specific and functional approaches to clarify the role of IL-17 signaling in the therapeutic actions of SCF+G-CSF and its broader contribution to immune modulation in AD pathogenesis. Our recent scRNA-seq study further highlights the importance of cell-specific immune profiling for defining treatment-associated immunomodulatory effects and facilitating the interpretation of neuroimmune transcriptomic changes that we hypothesize occur following SCF+G-CSF treatment.

### 3.6 SCF+G-CSF downregulates EGFR1, interleukin signaling, and MMP16: potential mechanisms underlying reduced AD pathology

STRING v12.0 interaction analysis identified *Cbl* and *Stam2* (signal transducing adaptor molecule) as functionally associated genes that were downregulated following SCF+G-CSF treatment (Figures 4, 5 and Table 2). Both genes participate in the epidermal growth factor (EGF)-related signaling pathways (Endo et al., 2000; Thien and Langdon, 2001). STAM2 is ubiquitously expressed and interacts with Janus kinases (JAKs), whose activation is regulated by cytokines, including IL-2 and GM-CSF (Endo et al., 2000). Through receptor-associated tyrosine phosphorylation, these signaling pathways regulate downstream targets involved in DNA synthesis and cell proliferation (Endo et al., 2000).

**TABLE 2:** SCF+G-CSF-induced pathway enrichment in the brains of aged APP/PS1 mice.

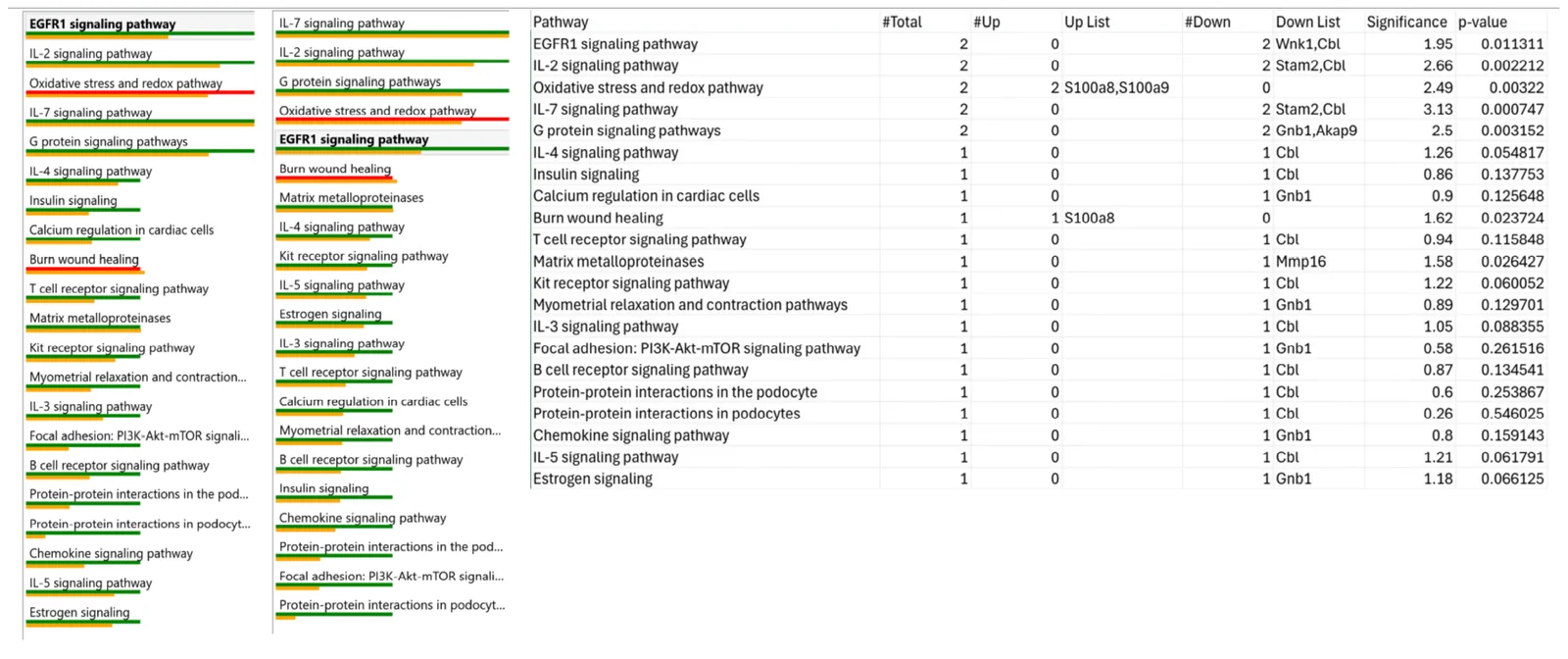

Yellow bars represent the degree of pathway enrichment. Green bars indicate the presence of SCF+G-CSF-downregulated differentially expressed genes (DEGs), whereas red bars indicate the presence of SCF+G-CSF-upregulated DEGs. The two leftmost tables show pathway enrichment organized by the number of genes involved and by statistical significance, respectively. The rightmost table lists the enriched pathways, their corresponding significance values, and the numbers of upregulated (#Up) and downregulated (#down) DEGs associated with each pathway. Data were analyzed using the Transcriptome Analysis Console (TAC).

In parallel with these network-level changes, SCF+G-CSF treatment was associated with downregulation of IL-2, IL-4, IL-7, and epidermal growth factor receptor 1 (EGFR1) in the brains of aged APP/PS1 mice (Figure 5 and Table 2). EGFR1 is a well-characterized member of the receptor tyrosine kinase family and has been extensively implicated in cancer biology (Jayaswamy et al., 2023). Emerging evidence further suggests that EGFR overactivation contributes to AD pathogenesis (Romano and Bucci, 2020). Consistent with this evidence, preformed amyloid fibrils have been reported to promote EGFR aggregation in the brain (Tavassoly and Tavassoly, 2021), and EGFR signaling has been implicated in amyloid-associated pathology and tau phosphorylation in AD models (Jayaswamy et al., 2023). Together, these findings raise the possibility that SCF+G-CSF-induced downregulation of EGFR1 may contribute to its therapeutic effects by attenuating EGFR signaling associated with AD pathology.

Collectively, our transcriptomic findings, together with previous studies, suggest that SCF+G-CSF may modulate cytokine- and growth factor-associated signaling networks relevant to AD pathology. However, the functional contribution of STAM2 to these signaling networks and its role in AD require further experimental investigation.

Additionally, SCF+G-CSF treatment was associated with downregulation of the matrix metalloproteinase 16 (MMP16) pathway, which may further contribute to reduced AD neuropathology (Figure 5 and Table 2). Matrix metalloproteinases (MMPs) are zinc- and calcium-dependent enzymes that degrade components of the extracellular matrix (ECM), including collagen, elastin, gelatin, and glycoproteins (Wang et al., 2014). The ECM provides structural and biochemical support to neurons and serves as a protective scaffold within the neural microenvironment. Under pathological conditions, MMPs contribute to inflammation and neurodegeneration by remodeling the perineuronal environment and altering the distribution of neurotransmitter and growth factor receptors (Wang et al., 2014). Members of the MMP family have been detected in senile plaques and neurofibrillary tangles in the hippocampus and cortex, and their expression is elevated in AD patients, where it is associated with cognitive decline (Wang et al., 2014). Based on these findings, we propose that SCF+G-CSF-induced downregulation of MMP16 may preserve extracellular matrix integrity and thereby contribute to reduced AD neuropathology. Preservation of ECM integrity may, in turn, reduce neuronal vulnerability and support neuronal function, although these possibilities remain to be experimentally validated.

### 3.7 Metal sequestration and immune modulation may contribute to reduced AD neuropathology following SCF+G-CSF treatment

The role of metal ion dyshomeostasis in AD neuropathology has received increasing attention. Emerging evidence suggests that metal ions, including manganese, copper, and iron not only promote amyloid and tau aggregation and tau phosphorylation but also increase the production of reactive oxygen species, thereby contributing to oxidative stress (Chen et al., 2023; Liu et al., 2022; Tjalkens et al., 2017). In addition, metal-dependent cell death pathways, including ferroptosis (iron-dependent) and cuproptosis (copper-dependent), have been implicated in AD pathology and may contribute to neuroinflammation, neuronal loss, and cognitive decline.

Evidence linking impaired metal ion homeostasis to AD-associated neuroinflammation has led to the proposal that metal chelators may represent potential therapeutic agents (Chen et al., 2023; Liu et al., 2022). In addition to promoting inflammation and protein aggregation, metal ions contribute to oxidative stress, neuronal loss, tau hyperphosphorylation, neurofibrillary tangle formation, and free radical generation, particularly through copper-amyloid complexes that can cause severe cellular damage (Liu et al., 2022).

Although the metal ion hypothesis of AD remains under investigation, our findings provide additional support for its potential relevance to both AD pathology and the therapeutic effects of SCF+G-CSF treatment in aged APP/PS1 mice. Our pathway analysis identified more than 400-fold enrichment of the *metal sequestration by antimicrobial proteins* pathway (Figure 6). STRING v12.0 analysis further demonstrated strong functional associations among four co-expressed genes (*S100a8*, *S100a9*, *Ltf*, and *Lcn2*) involved in this pathway (Figure 4). Together, these findings suggest that SCF+G-CSF may promote metal sequestration through upregulation of metal-binding proteins, thereby limiting the availability of metal ions implicated in AD-associated pathological processes. This proposed mechanism may contribute to the therapeutic effects of SCF+G-CSF by reducing metal ion-mediated oxidative stress, neuroinflammation, and protein aggregation in aged APP/PS1 mice (Guo et al., 2020).

In addition to metal ion dyshomeostasis, our findings suggest that dysregulation of intracellular and extracellular transport pathways may also contribute to AD neuropathology. Rho GTPases, members of the RAS superfamily, regulate intracellular trafficking, cytoskeletal organization, and vesicular transport and have increasingly been implicated in AD pathogenesis (Aguilar et al., 2017). Dysfunction of Rho GTPase signaling has been associated with Aβ plaque formation, neurofibrillary tangle development, synaptic dysfunction, and neuronal injury in experimental models of AD (Aguilar et al., 2017). Interestingly, SCF+G-CSF treatment enriched several Rho GTPase-associated signaling pathways, including pathways involved in NADPH oxidase regulation (Figures 4, 6 and Supplementary Figure S4). NADPH oxidase participates in innate immune responses and oxidative signaling and has also been implicated in neuroinflammation and amyloid-associated pathology in AD (Aguilar et al., 2017). In addition, Rho GTPase signaling regulates actin polymerization, synapse formation, and neuronal plasticity (Aguilar et al., 2017). Together, these findings raise the possibility that SCF+G-CSF-induced enrichment of Rho GTPase signaling (Supplementary Figure S4) may contribute to treatment-associated immunomodulation and adaptive cellular responses in aged APP/PS1 mice.

Additionally, two upstream regulators of Rho GTPase signaling, Ras and EGFR, have been implicated in AD pathology (Aguilar et al., 2017). In the present study, EGFR1 was downregulated following SCF+G-CSF treatment (Figure 5). Together with the enrichment of Rho GTPase-associated signaling pathways, these findings suggest that SCF+G-CSF may modulate interconnected signaling networks involved in neuroinflammation, cellular stress responses, and neuronal dysfunction. Whether EGFR1 downregulation directly influences Rho GTPase-mediated tau pathology remains to be determined.

### 3.8 SCF+G-CSF may counteract widespread transcriptional dysregulation in aged APP/PS1 mice

Given that our previous study demonstrated that SCF+G-CSF treatment mitigated AD neuropathology in aged APP/PS1 mice (Guo et al., 2020), we sought to perform transcriptomic analysis to determine how advanced-stage AD alters gene expression in the brains of aged APP/PS1 mice. To accomplish this, we compared gene chip (microarray) data from 17 to 18-month-old APP/PS1 mice with age-matched WT mice. Both groups received subcutaneous vehicle injections for 12 consecutive days and were euthanized on day 13. RNA extracted from the frontal lobe was subjected to transcriptomic analysis (Figure 1). Again, we specifically selected aged APP/PS1 mice because they represent a more advanced stage of AD pathology that more closely resembles the clinical condition in patients with AD.

A list of DEGs in vehicle-treated APP/PS1 (CON) mice compared with age-matched WT mice is shown in Figure 7 and Table 3. Hierarchical clustering of these DEGs is presented in the heat map shown in Figure 8, where red indicates upregulated genes and blue indicates downregulated genes. Transcriptomic analysis using Thermo Fisher Scientific’s Transcriptome Analysis Console identified 89 significantly differentially expressed genes (≥2-fold change) among 45,037 detected genes (0.2%). Of these 89 genes, 85 (95.5%) were upregulated, and 4 (4.5%) were downregulated in aged APP/PS1 mice compared with age-matched WT controls (Figure 7A).

**TABLE 3:** Pathway enrichment in aged APP/PS1 mice (CON) versus age-matched WT mice in the absence of SCF+G- CSF.

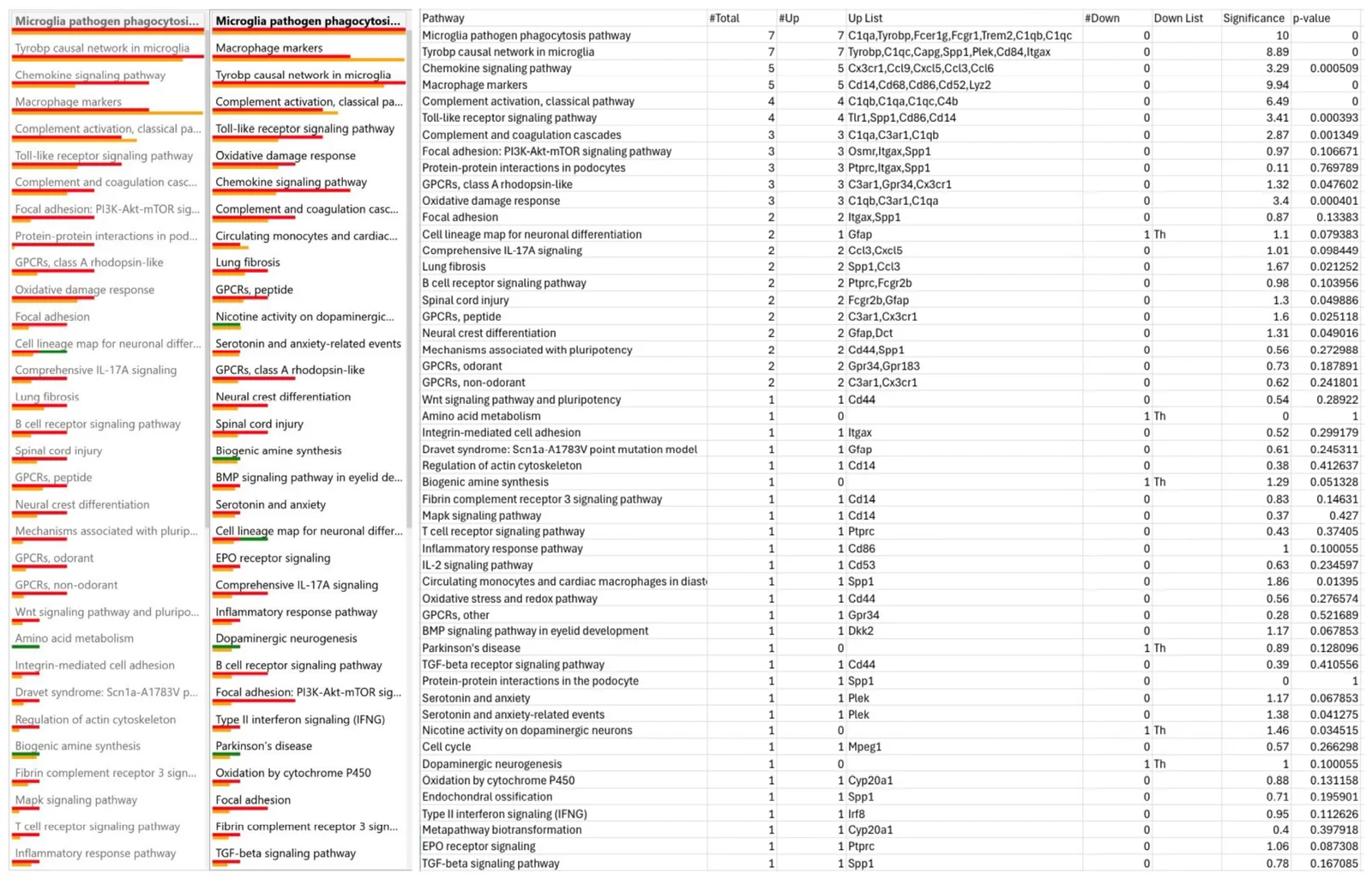

Pathway enrichment in aged APP/PS1 mice (CON) compared with age-matched WT mice in the absence of SCF+G-CSF treatment. Yellow bars represent the degree of pathway enrichment. Red bars indicate the presence of upregulated differentially expressed genes (DEGs), whereas green bars indicate the presence of downregulated DEGs. The two leftmost tables show pathway enrichment organized by the number of genes involved and by statistical significance, respectively. The rightmost table lists the enriched pathways, their corresponding significance values, and the numbers of upregulated (#Up) and downregulated (#down) DEGs associated with each pathway. Data were analyzed using the Transcriptome Analysis Console (TAC). CON: vehicle-injected APP/PS1 mice. WT: vehicle-injected wild-type mice.

**FIGURE 7:**
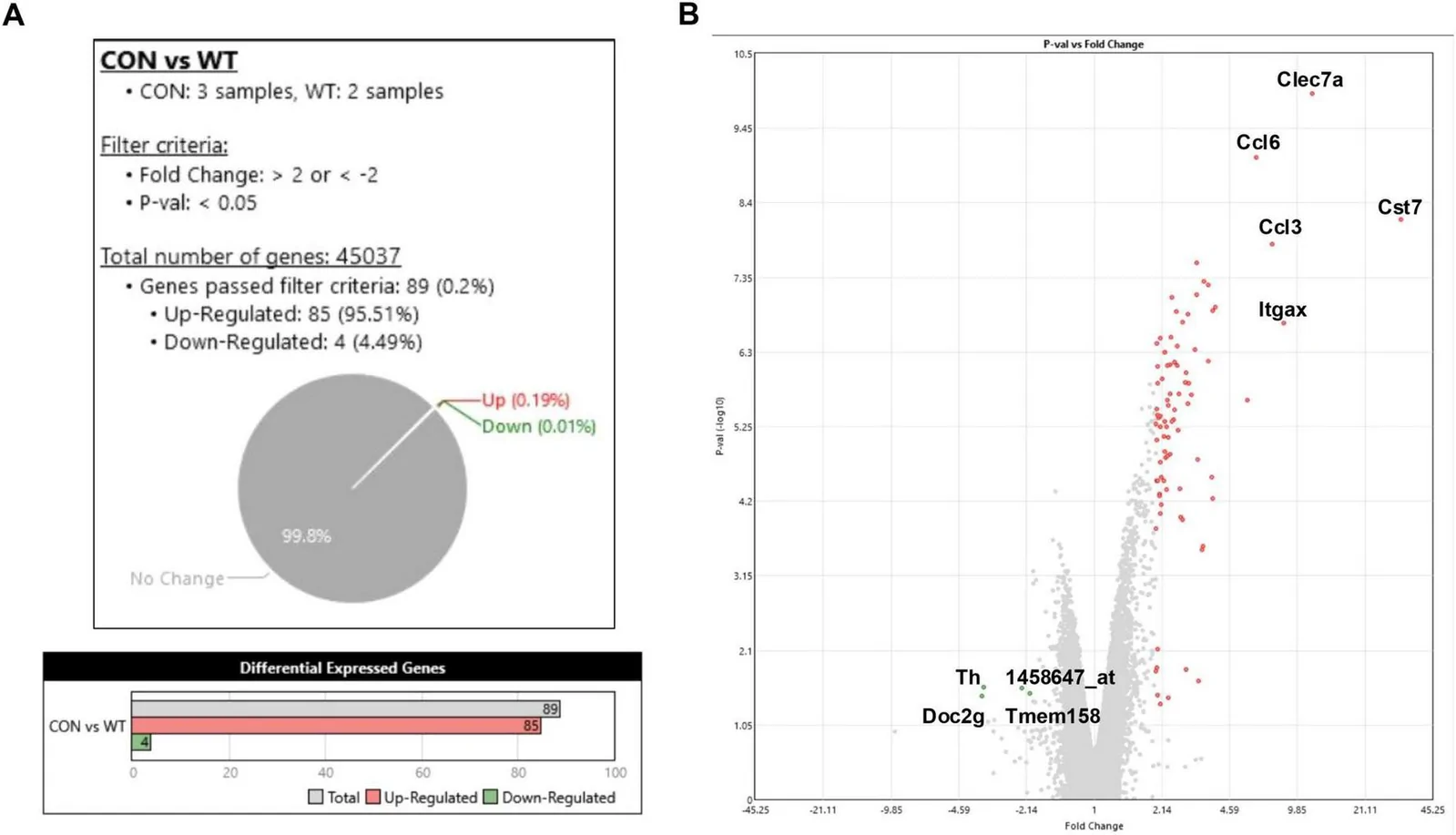
Summary and volcano plot of gene expression differences between aged APP/PS1 and aged WT mice analyzed using the Transcriptome Analysis Console (TAC). **(A)** A total of 89 differentially expressed genes (DEGs) are identified in the brains of aged APP/PS1 mice compared with age-matched WT controls, with more than 95% of the DEGs upregulated. This finding indicates that gene upregulation predominates in untreated aged APP/PS1 mice. **(B)** The five most significantly upregulated and downregulated DEGs are labeled and bolded. Genes labeled only by numerical probe set identifiers do not have annotated gene names or symbols. CON, aged APP/PS1 mice injected with vehicle; WT, age-matched wild-type mice.

**FIGURE 8:**
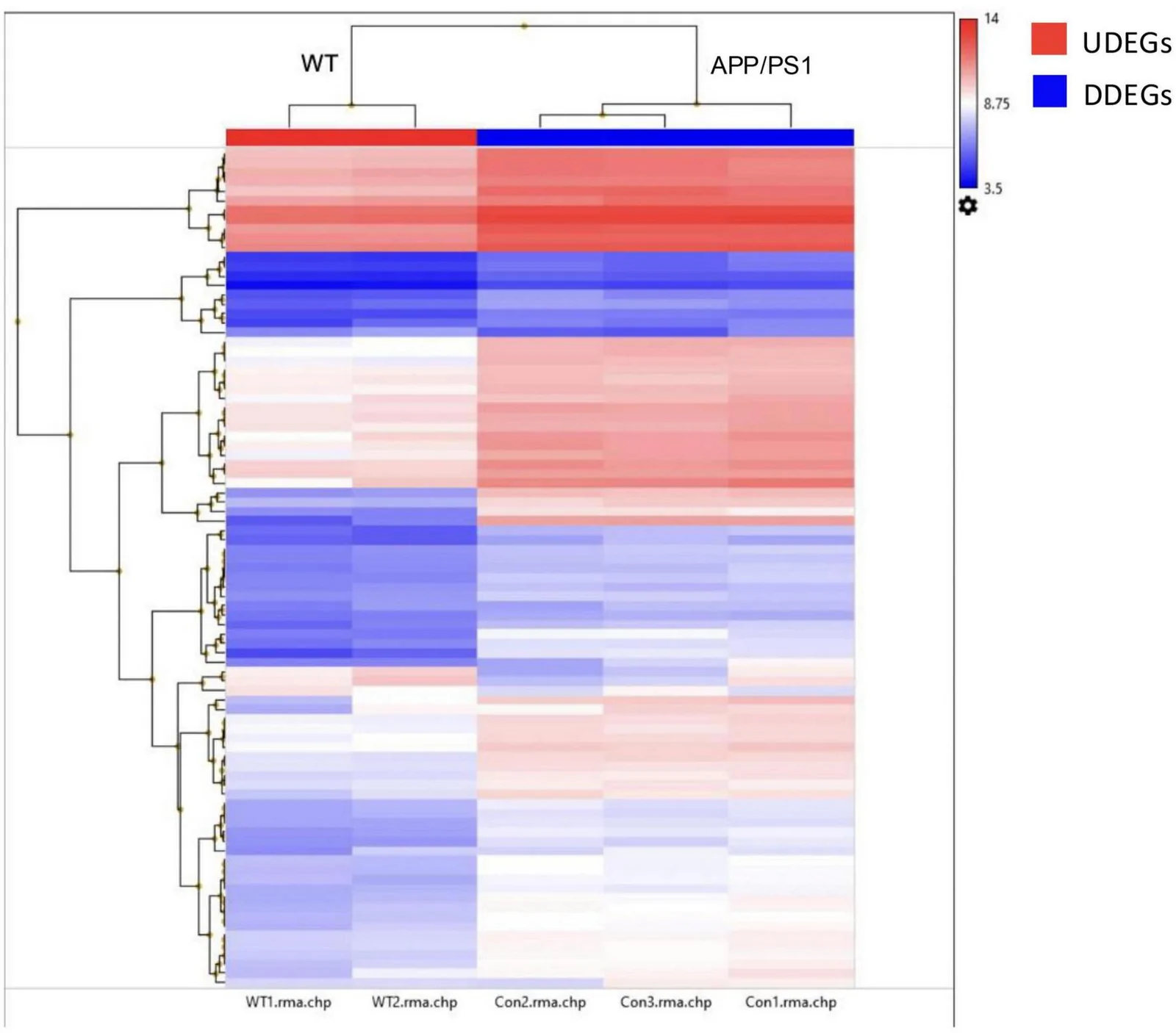
Heat map showing hierarchical clustering of differentially expressed genes (DEGs) in aged WT and APP/PS1 mice. Normalized gene expression is displayed on a log-scaled gradient, where darker colors indicate higher expression levels and lighter colors indicate lower expression levels. Upregulated DEGs are marked in red, and downregulated DEGs in blue. The heat map was generated using the Transcriptome Analysis Console (TAC). CON, aged APP/PS1 mice injected with vehicle; WT, age-matched wild-type mice.

In contrast, SCF+G-CSF treatment downregulated more than 70% of treatment-associated DEGs, with only approximately 29.6% of DEGs being upregulated in aged APP/PS1 mice (Figure 2). Together with the chromosomal distribution analysis (Supplementary Figure S5), these findings suggest that untreated aged APP/PS1 mice exhibit widespread transcriptional dysregulation compared with age-matched WT mice. Conversely, the marked reduction in upregulated genes following SCF+G-CSF treatment suggests that the treatment may partially restore transcriptional control disrupted during AD progression.

### 3.9 Transcriptional analysis reveals upregulation of genes associated with innate immune pathways and microglial activation in aged APP/PS1 mice

Next, we sought to identify the biological pathways associated with our DEG dataset. Interestingly, we found that biological functions relevant to AD pathology and the experimental groups were already upregulated in untreated (vehicle-injected) aged APP/PS1 mice compared with healthy age-matched WT controls (Figures 9A–D, 10A–D).

**FIGURE 9:**
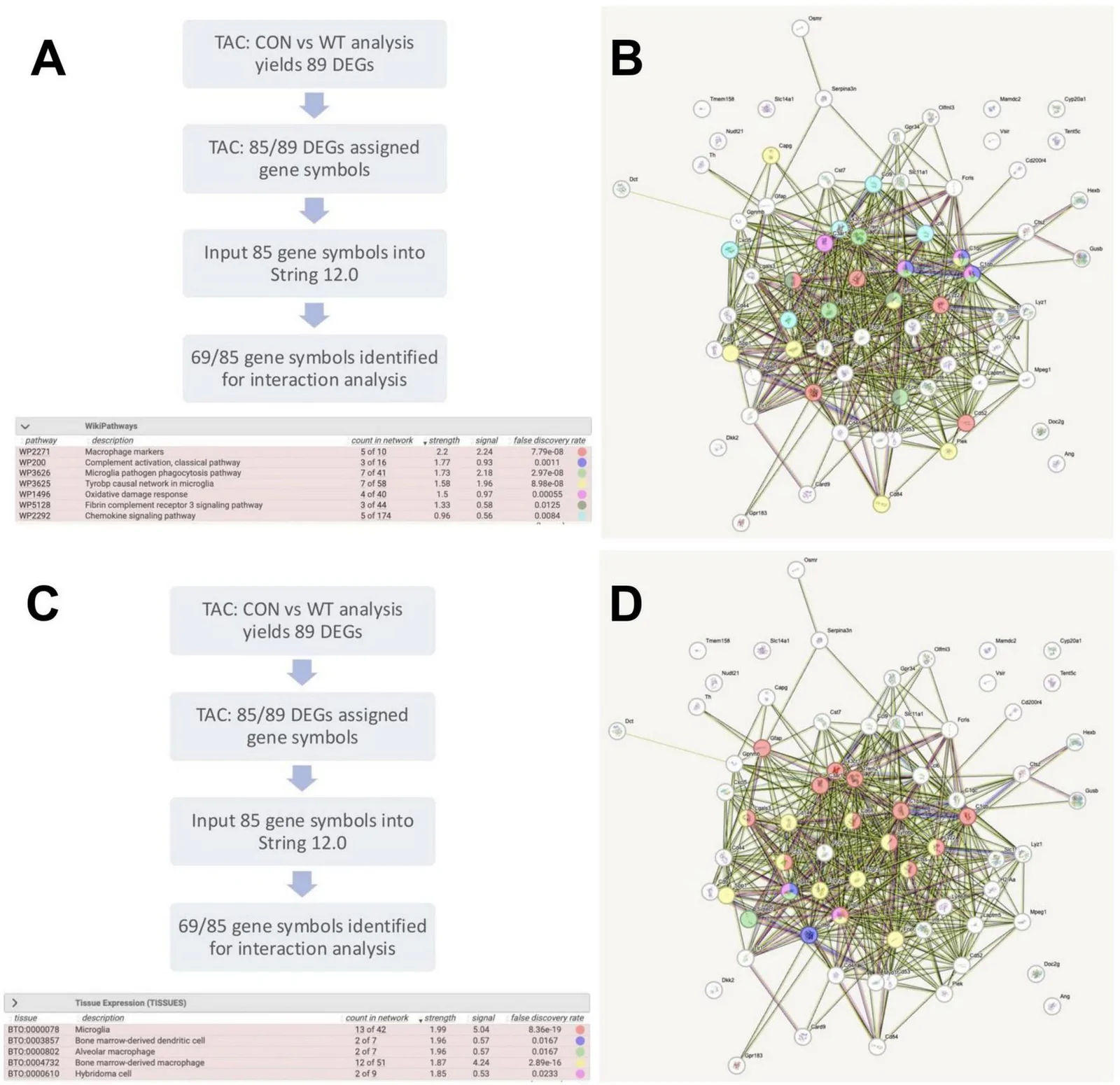
STRING v12.0 pathway analysis of differentially expressed genes (DEGs) highlights enrichment in macrophage marker-associated and innate immune system pathways in the brains of aged APP/PS1 mice. **(A,C)** Transcriptome Analysis Console (TAC) identified 89 DEGs, of which 85 had known gene names. Among these 85 genes, 69 genes were identified by STRING v12.0 interaction analysis. Enriched functional clusters are color-coded by pathway type. **(B)** A prominent gene cluster shows enrichment of macrophage marker-associated pathways and innate immune responses, including complement and phagocytosis pathways, suggesting enhanced immune signaling in the aged APP/PS1 mouse brain. **(D)** Another enriched gene cluster is associated with pathways expressed by microglia and bone marrow-derived macrophages, suggesting contributions from both central and peripheral immune cell populations in the AD state. CON, aged APP/PS1 mice injected with vehicle; WT, age-matched wild-type mice.

**FIGURE 10:**
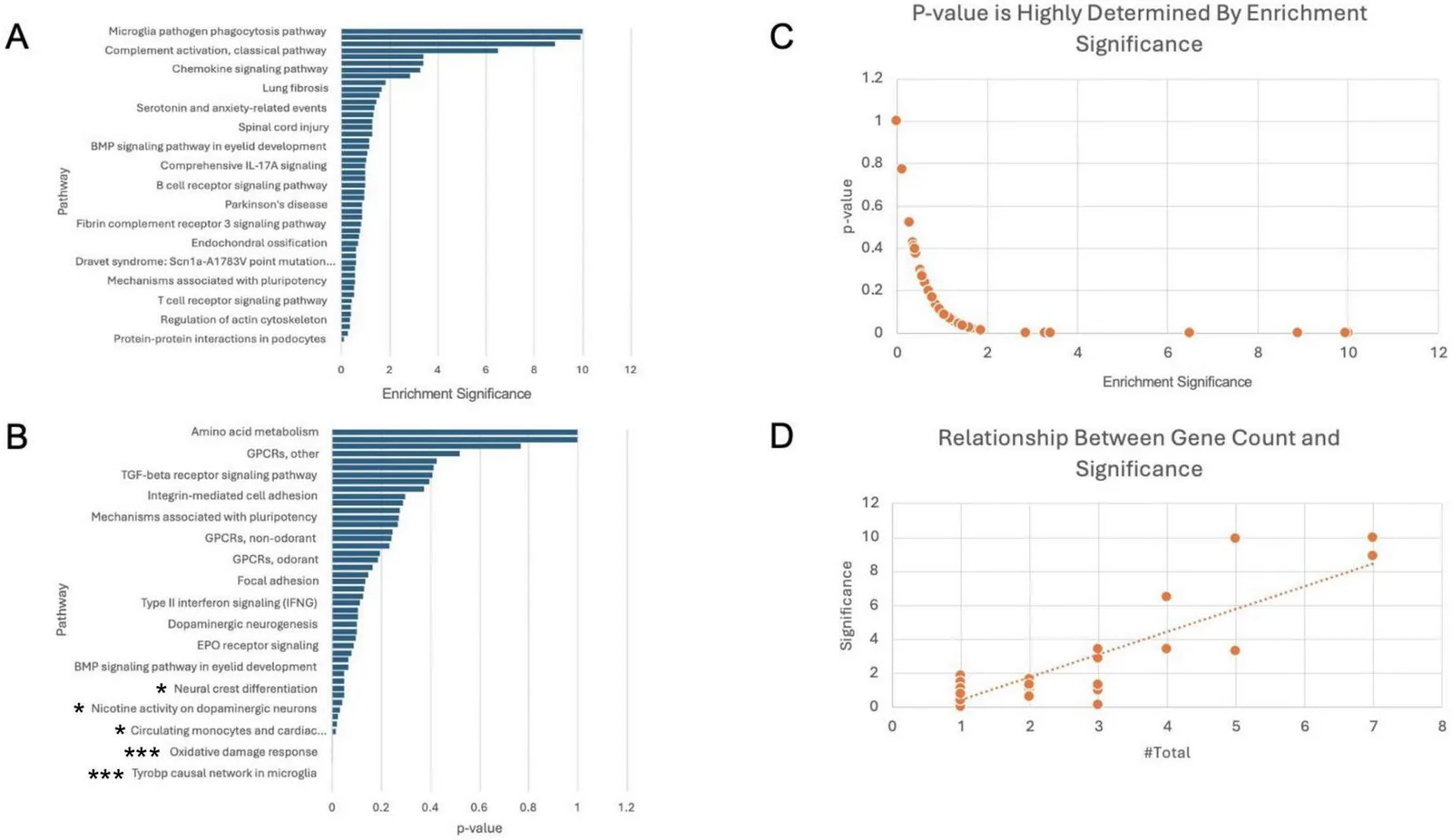
Transcriptome analysis console (TAC) pathway enrichment analysis highlights microglial, innate immune, and oxidative stress-related pathways in aged APP/PS1 mouse brains. **(A)** Pathway enrichment analysis ranked by significance highlights microglial- and immune-related pathways associated with AD pathology. **(B)** Pathway enrichment analysis ranked by *p*-value identifies highly significant pathways, including the Tyrobp causal network in microglia and oxidative damage response; *p < 0.05, ***p < 0.001. **(C)** Statistical analysis shows an inverse exponential relationship between *p*-value and enrichment significance. **(D)** A positive correlation is observed between the number of differentially expressed genes (DEGs) and pathway enrichment significance, indicating that more significantly enriched pathways are associated with a greater number of DEGs.

Both STRING v12.0 and TAC analyses identified a large gene cluster with the highest enrichment in macrophages, bone marrow-derived macrophages/dendritic cells, and microglia, as well as pathways involving these cell types and the innate immune system, including microglia pathogen phagocytosis, complement activation (classical pathway), and chemokine signaling (Figures 9, 10). These findings led us to hypothesize that the immune system in aged APP/PS1 mice is highly activated in response to AD neuropathology. This suggests that endogenous immune and inflammatory pathways are naturally engaged in an attempt to counter AD-associated pathology in the absence of therapeutic intervention.

Enriched biological functions in aged APP/PS1 mice, compared with age-matched WT mice, included microglial phagocytosis, complement activation (particularly the classical pathway), and chemokine signaling (Figure 10A and Table 3). Among these, the most significantly enriched pathway, ranked by *p* value, was the TYROBP causal microglial network, which included 7 upregulated DEGs: *Tyrobp, C1qc, Capg, Spp1, Plek, Cd84*, and *Itgax* (Figure 10B and Table 3). Other significantly enriched pathways included responses to oxidative damage, as well as transcriptional signatures associated with circulating monocytes and cardiac macrophages linked to diastolic dysfunction (Figure 10B and Table 3).

TYROBP (tyrosine kinase-binding protein) is a transmembrane immune signaling adaptor that is highly expressed in microglia and has been implicated in AD-associated neuroinflammatory signaling (Ma et al., 2015). It plays a central role in signal transduction mediated by microglia, macrophages, dendritic cells, and osteoclasts (Ma et al., 2015). Evidence suggests that TYROBP promotes microglial phagocytosis of amyloid plaques and neuronal debris while also modulating inflammatory responses by suppressing microglia-mediated cytokine production (Ma et al., 2015). A previous study using a PSEN1 mutant mouse model of AD reported that *Tyrobp* was preferentially expressed in hippocampal microglia, and that *Tyrobp*-deficient mice exhibited slower cognitive decline, accompanied by reduced levels of proinflammatory cytokines (TNF-α, IL-1β, IL-6) and fewer astrocytes and microglia in the hippocampus (Li R. et al., 2022).

Taken together, our findings, together with previous studies, suggest that TYROBP upregulation in aged APP/PS1 mice may represent a compensatory response to increased amyloid burden. Interestingly, *Tyrobp* was not significantly differentially expressed in SCF+G-CSF-treated aged APP/PS1 mice compared with vehicle-treated aged APP/PS1 mice. We therefore hypothesize that excessive compensatory microglial activation, including that mediated by TYROBP, may become maladaptive during AD progression. The absence of *Tyrobp* upregulation following SCF+G-CSF treatment may instead reflect reduced neuroinflammatory stress or a shift toward a more regulated microglial activation state. We propose that SCF+G-CSF promotes a more balanced immune response rather than excessive compensatory microglial activation. These hypotheses, however, require validation in future studies.

### 3.10 Functions and implications of the top 5 upregulated DEGs in aged APP/PS1 mice

*Cst7. Cst7* was markedly upregulated 31.73-fold in aged APP/PS1 mice compared with age-matched WT mice (Figure 7B and Table 4). Also known as cystatin F, *Cst7* is a cysteine protease inhibitor localized to the endolysosome and involved in the regulation of immune cell cytotoxicity (Kos et al., 2018). It is highly expressed in microglia and/or monocyte-derived macrophages (Daniels et al., 2023; Li et al., 2024; Ma et al., 2011). Elevated levels of dimerized cystatin F have been detected in the blood of AD patients (Li et al., 2024), and the protein is known to bind Aβ, interfering with its recognition and uptake by monocytes (Li et al., 2024). This impaired clearance may contribute to amyloid accumulation and the associated inflammatory response. We postulate that the marked upregulation of *Cst7* may contribute to the activation of phagocytic pathways, such as those regulated by TYROBP, as a compensatory response to the increased Aβ burden in aged APP/PS1 mice. Given its reported roles in impaired Aβ clearance and immune dysregulation, it is not surprising that *Cst7* upregulation is associated with worsening clinical manifestations in AD. However, whether *Cst7* directly drives AD pathology or instead reflects a compensatory immune response remains unclear and warrants further investigation.

**TABLE 4:** DEGs in aged APP/PS1 mice (CON) versus aged matched WT mice in the absence of SCF+G-CSF.

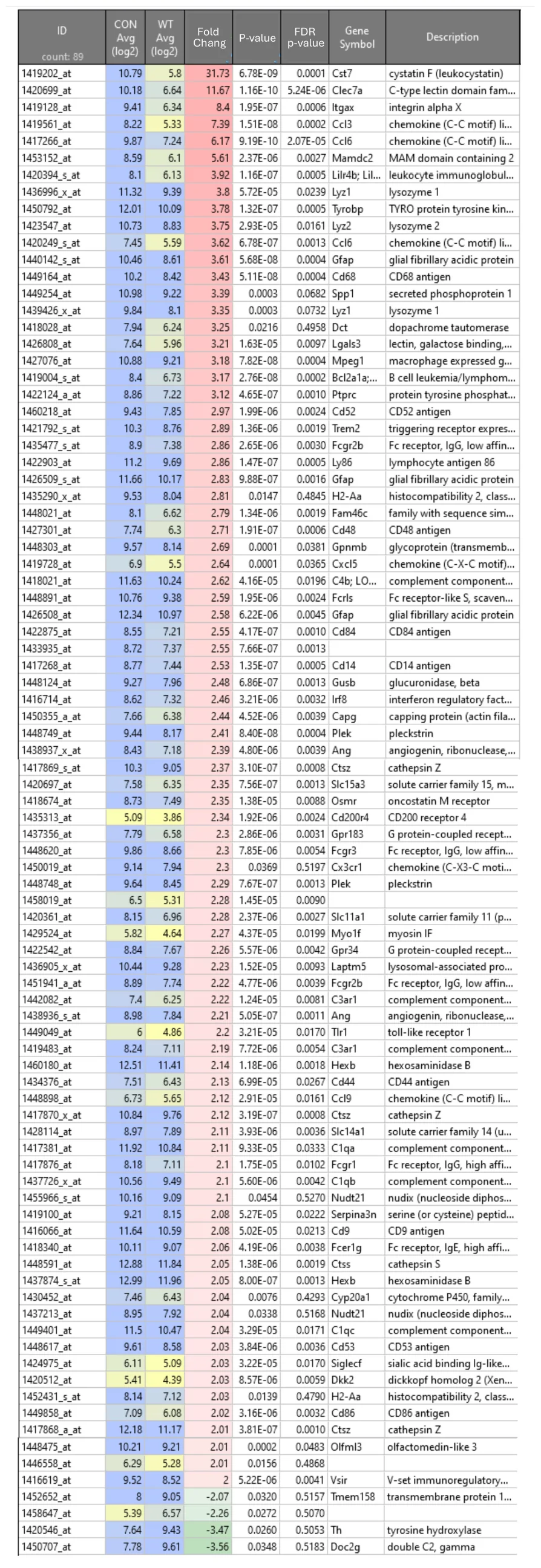

Red-highlighted fold changes represent upregulated differentially expressed genes (DEGs), whereas green-highlighted fold changes represent downregulated DEGs in aged APP/PS1 mice compared with wild-type (WT) mice. Color gradients indicate the relative magnitude of the fold changes within each group. CON and WT averages represent the microarray signal intensities for each gene. Data were analyzed using the Transcriptome Analysis Console (TAC). CON: vehicle-injected APP/PS1 mice. WT: vehicle-injected wild-type mice.

*Clec7a. Clec7a* was upregulated 11.67-fold in aged APP/PS1 mice compared with age-matched WT mice (Figure 7B and Table 4). *Clec7a* encodes the Dectin-1 protein, a receptor expressed on macrophages and innate immune cells that recognizes β-glucans in fungal cell walls and activates macrophage responses (Brown et al., 2002). It also signals through DAP12/TYROBP-associated pathways that converge on spleen tyrosine kinase (SYK)-dependent immune signaling cascades, supporting its role in innate immune receptor-mediated signaling during microglial responses to amyloid pathology (Wang et al., 2024; Yao et al., 2019).

These findings further support the concept that AD pathology involves dysregulation of the innate immune system. Our data suggest that increased amyloid burden in aged APP/PS1 mice is associated with altered innate immune activation (Figures 9, 10A,B). This highlights the complexity of targeting *Clec7a* as a therapeutic target in AD. Its precise role in AD pathogenesis remains to be determined through future studies.

*Itgax. Itgax* was upregulated 8.40-fold in aged APP/PS1 mice compared with age-matched WT mice (Figure 7B and Table 4). Also known as integrin subunit alpha X (CD11c), *Itgax* is a marker associated with microglial activation states and disease-associated microglial populations in neurodegenerative diseases (Lopes et al., 2024). Previous studies have shown that elevated *Itgax* expression is associated with cognitive decline and more severe AD, and it is often observed alongside other disease-associated microglial markers, such as *Spp1* (Lopes et al., 2024).

In the present study, *Itgax* gene expression was significantly higher in the brains of aged APP/PS1 mice than in age-matched WT controls. Given the advanced neuropathological stage of aged APP/PS1 mice, *Itgax* upregulation is not unexpected. Importantly, the absence of *Itgax* upregulation in SCF+G-CSF-treated aged APP/PS1 mice supports the therapeutic potential of SCF+G-CSF, suggesting that the treatment may modulate the expression of microglia activation markers and influence disease-associated microglial states. The precise mechanism, however, requires further validation.

*Ccl3 and Ccl6. Ccl3* and *Ccl6* were upregulated 7.39- and 6.17-fold, respectively, in aged APP/PS1 mice compared with age-matched WT controls (Figure 7B and Table 4). *Ccl3* and *Ccl6* encode the chemokine ligands CCL3 and CCL6, which act as chemoattractants for microglia and contribute to immune cell recruitment during neuroinflammatory responses (Cudaback et al., 2015; Nakagawa et al., 2019). CCL3 secretion is modulated by APOE in astrocytes (Cudaback et al., 2015), while CCL6 may reduce detrimental neuron-astrocyte interactions, thereby protecting neurons from glutamate-induced excitotoxicity (Nakagawa et al., 2019). Additionally, CCL6 has been implicated in promoting M2 macrophage polarization at inflammatory sites through activation of the PI3K-Akt signaling pathway (Feng et al., 2023).

Altogether, the upregulation of *Ccl3* and *Ccl6* suggests a potential association with chemokine-mediated immune signaling in the diseased brain, although their precise functional roles in AD remain to be determined.

### 3.11 Functions and implications of the downregulated DEGs in aged APP/PS1 mice

*Doc2g. Doc2g* was downregulated 3.56-fold in aged APP/PS1 mice compared with age-matched WT controls (Figure 7B and Table 4). *Doc2g* encodes the double C2 domain-containing protein gamma (DOC2G), a member of the double C2 domain family involved in calcium-dependent binding to phospholipid membranes and regulation of synaptic vesicle exocytosis (Friedrich et al., 2010). While other family members, such as DOC2A and DOC2B, facilitate neurotransmitter release through phospholipid interactions, DOC2G is unique in that it does not bind phospholipids (Fukuda and Mikoshiba, 2000).

Synaptic dysfunction and abnormalities in neurotransmission are hallmarks of AD, particularly involving glutamate and acetylcholine, which play essential roles in synaptic transmission, learning, and memory (Francis, 2003). Although the precise function of *Doc2g* remains under investigation, the observed downregulation of this DOC2 family member may reflect synaptic alterations in vehicle-treated aged APP/PS1 mice.

*Th. Th* was downregulated 3.47-fold in aged APP/PS1 mice compared with age-matched WT controls (Figure 7B and Table 4). *Th* encodes tyrosine hydroxylase (TH), the rate-limiting enzyme in dopamine (DA) synthesis through the conversion of tyrosine to L-DOPA. Deficiency of TH is classically associated with Parkinson’s disease, in which degeneration of dopaminergic neurons in the substantia nigra and loss of neuromelanin contribute to motor dysfunction (Nagatsu et al., 2022). This process is associated with oxidative stress, impaired autophagy, and neuroinflammation (Nagatsu et al., 2022).

Although the role of *Th* in AD remains under investigation, reduced TH expression has also been reported in AD brains (Aliev et al., 2014). Aβ-mediated damage to noradrenergic neurons, particularly those projecting to the hippocampus, has been implicated in synaptic dysfunction, neuronal loss, and cognitive decline (Aliev et al., 2014). Thus, the observed downregulation of *Th* in aged APP/PS1 mice may be associated with increased amyloid burden and neuroinflammatory stress, further supporting a link between AD and neurotransmission abnormalities. These findings underscore the need for continued investigation into monoaminergic dysfunction in AD pathogenesis.

*Tmem158. Tmem158* was downregulated 2.07-fold in aged APP/PS1 mice compared with age-matched WT controls (Figure 7B and Table 4). *Tmem158*, which encodes transmembrane protein 158, has been implicated in promoting cancer cell proliferation, migration, and survival through activation of STAT3-associated signaling pathways in glioma (Li J. et al., 2022) and TGF-β-associated signaling pathways in breast and pancreatic cancer models (Fu et al., 2020; Tong et al., 2022). Although the role of *Tmem158* in AD remains poorly understood, its downregulation in the present study may be associated with reduced activity of growth- and survival-related signaling pathways. Whether these changes contribute to neurodegenerative processes in AD remains to be determined.

### 3.12 Additional functional analyses reveal glycosaminoglycan degradation, immune dysregulation, and disease-associated pathways in aged APP/PS1 mice

ShinyGO 0.77 analysis revealed that glycosaminoglycan (GAG) degradation was the most significantly enriched biological function process, showing nearly 20-fold enrichment, in aged APP/PS1 mice compared with age-matched WT controls (Supplementary Figure S6). GAGs are major components of proteoglycans (Fukuchi et al., 1998; Snow et al., 2021), particularly those containing heparan sulfate (HS) polysaccharide. Notably, excessive HS GAG accumulation has been shown to promote the formation of Maltese/star-like Aβ_1–40_ deposits (Snow et al., 2021) and tau aggregation into paired helical filaments (Fukuchi et al., 1998; Snow et al., 2021), both of which are resistant to degradation and ultimately accumulate in the cortex and hippocampus, thereby contributing to AD pathology.

Interestingly, GAG degradation, rather than GAG biosynthesis, was enriched in the brains of aged APP/PS1 mice in the absence of SCF+G-CSF treatment, suggesting a potential compensatory response aimed at limiting pathological amyloid and tau accumulation. This finding challenges the view that the aged APP/PS1 mouse brain represents a functionally inert or irreversible AD state. Instead, it suggests that even during advanced stages of AD, the brain may retain endogenous responses that attempt to counter disease-associated pathology (Figures 9, 10A,B). In this context, our previous studies suggest that SCF+G-CSF treatment may further enhance these reparative responses, including stimulation of macrophage-mediated amyloid clearance (Gardner et al., 2024; Guo et al., 2020; Li et al., 2011).

Consistent with AD pathology, our analysis also identified nearly 15-fold enrichment of the complement cascade activation pathway (Supplementary Figure S6A). Fibrillar amyloid is known to activate both the classical and alternative complement pathways through interactions with C1q and C3, ultimately leading to the formation of the membrane attack complex (MAC), which damages neuronal membranes and promotes neuroinflammation (Kolev et al., 2009). These findings are consistent with previous studies and further support the involvement of innate immune activation in chronic AD.

Additionally, aged APP/PS1 mice without SCF+G-CSF treatment exhibited enrichment of pathways associated with hematologic and bone disorders, as well as infectious and inflammatory disease processes (Supplementary Figure S6A). In contrast, SCF+G-CSF-treated aged APP/PS1 mice showed upregulation of genes associated with antimicrobial defense and immune regulation, as discussed earlier. Previous studies have demonstrated that SCF+G-CSF treatment promotes brain repair and neurostructural remodeling in animal models of stroke (Cui et al., 2013; Piao et al., 2009; Zhao et al., 2007a; Zhao et al., 2007b). SCF+G-CSF treatment has also been used clinically to mobilize peripheral hematopoietic stem cells for the treatment of hematologic malignancies (Facon et al., 1999; Stiff et al., 2000).

Collectively, these observations suggest that examining diverse disease processes may provide additional insights into mechanisms influencing AD progression, resistance, or susceptibility, thereby supporting the development of new therapeutic strategies for AD.

## 4 Concluding remarks

AD is the leading cause of dementia worldwide, with an estimated $305 billion spent on the care of individuals aged 65 years and older with dementia in the United States in 2020 (Wong, 2020). Our previous studies demonstrated that combined SCF+G-CSF treatment reduces AD-related pathologies, including amyloid burden, aggregated tau accumulation, dendritic and synaptic loss, neuroinflammation (Guo et al., 2020; Li et al., 2011), and cognitive impairments in APP/PS1 mice (Li et al., unpublished observations). Bone marrow-derived macrophages have also been shown to participate in Aβ phagocytosis and clearance (Li et al., 2011).

SCF+G-CSF combination treatment has shown therapeutic potential in experimental stroke (Cui et al., 2013; Piao et al., 2009; Zhao et al., 2007a; Zhao et al., 2007b) and AD (Gardner et al., 2024; Guo et al., 2020; Li et al., 2011), as well as in promoting hematopoietic stem/progenitor cell recovery following chemotherapy (Facon et al., 1999; Stiff et al., 2000; To et al., 2003). Despite these encouraging findings, the molecular mechanisms underlying its therapeutic effects remain poorly understood. The goal of the present study was to use transcriptomic profiling by microarray analysis to investigate molecular changes associated with SCF+G-CSF treatment in aged APP/PS1 mice and to identify potential molecular pathways and targets for future investigation.

The present study employed an integrated transcriptomic analysis using multiple complementary bioinformatic platforms, including TAC, STRING v12.0, and ShinyGO 0.77. In addition, the use of the well-characterized aged APP/PS1 mouse model enabled characterization of transcriptomic changes associated with advanced AD pathology. By comparing transcriptomic profiles among SCF+G-CSF-treated APP/PS1 mice, vehicle control APP/PS1 mice, and age-matched WT controls, we characterized transcriptional changes associated with advanced AD pathology and their modulation by SCF+G-CSF treatment.

Nonetheless, the study has several limitations. First, the relatively small sample size is a common constraint in gene chip studies and warrants cautious interpretation of the findings. Second, the study was conducted exclusively in male mice, which may limit the generalizability of the results, given the well-recognized sex differences in AD pathology and immune responses.

In addition, because transcriptomic analysis was performed using bulk brain RNA, the cellular sources of the differentially expressed genes could not be determined directly. However, our recently published scRNA-seq analysis of brain CD11b^+^ cells from aged APP/PS1 mice demonstrated that SCF+G-CSF significantly increased the cerebral CD45^high^/CD11b^+^ phagocyte population and robustly upregulated *S100a8* and *S100a9* across CD11b^+^ cell clusters (Gardner et al., 2024). Furthermore, the majority of the top SCF+G-CSF-induced differentially expressed genes were enriched in S100a8/9-positive cells (Gardner et al., 2024), supporting the conclusion that the immune-related transcriptional signature identified in the present study is closely associated with SCF+G-CSF-induced immunomodulation of brain CD11b^+^ immune cells, including both resident microglia and peripherally derived myeloid cells. Nevertheless, additional studies using spatial transcriptomics, immunofluorescence, or lineage-tracing approaches will further define the cellular localization of these transcriptomic changes.

To strengthen the transcriptomic findings, *S100a8* and *S100a9*, the two most highly upregulated differentially expressed genes identified by the gene-chip analysis following SCF+G-CSF treatment, were selected for qRT-PCR validation because they represented the dominant treatment-induced immune-related transcriptional signature. Their upregulation was confirmed in an independent cohort of aged APP/PS1 mice and was further supported by our published scRNA-seq dataset (Gardner et al., 2024). Although additional validation of other differentially expressed genes, including *Lcn2*, *Ltf*, *Cbl*, and *Akap9*, as well as protein-level analyses, would further strengthen the study, these genes were not examined in the present work and therefore remain transcriptomic observations that warrant future investigation.

This study is also limited by its focus on a single brain region rather than a multi-region analysis. Transcriptomic profiling of whole-brain tissue can introduce substantial cellular and regional heterogeneity, potentially obscuring region-specific biological signals. Therefore, we analyzed the frontal cortex as a disease-relevant cortical region for this initial transcriptomic study. By 18 months of age, APP/PS1 mice exhibit widespread AD-like pathology involving both the neocortex and hippocampus, with substantial amyloid deposition and neuroinflammatory changes in the frontal cortex. In addition, the frontal cortex is increasingly recognized as an integral component of distributed memory networks that interact with hippocampal circuits to support learning and memory processes (de Sousa et al., 2026). Nevertheless, we acknowledge that the hippocampus is also a key brain region affected in AD and plays a critical role in cognitive dysfunction. It may also exhibit distinct transcriptomic responses to SCF+G-CSF treatment. Future studies comparing multiple brain regions, including the frontal cortex and hippocampus, will be important to define region-specific therapeutic effects and provide a more comprehensive understanding of the molecular mechanisms underlying SCF+G-CSF treatment.

Our transcriptomic analysis of upregulated and downregulated DEGs suggests that SCF+G-CSF treatment may promote metal ion sequestration through the induction of metal-binding proteins while also exerting immunomodulatory effects. The coordinated transcriptional changes further suggest that SCF+G-CSF may create an immune environment conducive to microglial activation, enhanced phagocytic activity, and amyloid localization (Figures 4, 6 and Supplementary Figures S2, S3). Together, these molecular changes may contribute to the therapeutic effects of SCF+G-CSF in aged APP/PS1 mice and provide a foundation for future mechanistic studies.

These mechanisms may include, but are not limited to, the coordinated upregulation and co-expression of a 6-member gene cluster (*S100a8*, *S100a9, Lcn2*, *Ngp, Ltf*, and *Camp*) (Figure 4). These findings suggest that inflammation, although often viewed as detrimental in AD, may also play an important role in initiating immune and microglial activation.

Accordingly, our study findings suggest that the conventional neuroinflammatory model of AD, which proposes that therapeutic approaches should broadly suppress inflammation, may require further refinement given the complexity of neuroinflammatory processes. Instead, our findings support the hypothesis that selected pro-inflammatory responses may represent important components of the innate immune system involved in modulating AD pathology. Importantly, the presence of S100A9 at amyloid plaques or the colocalization of S100A8/A9 with amyloid-β should not be interpreted as evidence of a causal role in AD pathology.

Based on the immune- and antimicrobial-related pathways enriched in our dataset, together with our previous findings demonstrating that SCF+G-CSF enhances the recruitment of bone marrow-derived macrophages involved in Aβ clearance (Li et al., 2011), we hypothesize that SCF+G-CSF treatment may promote the recruitment and/or activation of bone marrow-derived macrophages that contribute to Aβ clearance. We found that the IL-17 signaling pathway was highly enriched in SCF+G-CSF-treated APP/PS1 mice compared with vehicle control APP/PS1 mice. We further hypothesize that enrichment of the IL-17 signaling pathway may be associated with the coordinated upregulation of *S100a8, S100a9*, and *Lcn2* (Figure 4 and Supplementary Figure S2), thereby facilitating recruitment of innate and adaptive immune cells. In addition, S100a8 and S100a9 have been implicated in regulating immune responses, amyloid plaque aggregation (Cristovao and Gomes, 2019; Hagmeyer et al., 2019), and oxidative burst pathways (Markowitz and Carson, 2013), which contribute to the degradation of intracellular and extracellular debris.

Additionally, SCF+G-CSF treatment downregulated *EGFR1*, a receptor tyrosine kinase implicated in tumorigenesis (Romano and Bucci, 2020). This downregulation may contribute to the reduction of amyloid- and tau-associated pathology observed following SCF+G-CSF treatment (Figure 5). Although the role of EGFR1 signaling in AD remains incompletely understood, EGFR1 interacts with inflammatory mediators such as TNF-α and regulates neural stem cell proliferation within the subventricular zone (Romano and Bucci, 2020). Reduced EGFR1 signaling is associated with neurodegeneration and a shift toward glial rather than neuronal differentiation, underscoring the context-dependent functions of EGFR1 in the CNS (Romano and Bucci, 2020). Therefore, the biological consequences of SCF+G-CSF-induced *EGFR1* downregulation in AD require further investigation.

Our transcriptomic analysis also revealed that SCF+G-CSF treatment in aged APP/PS1 mice was associated with downregulation of key cytokine signaling pathways, including IL-7, IL-2, and IL-4, as well as MMP16-associated pathways (Figure 5). These findings suggest that SCF+G-CSF may modulate neuroinflammatory responses while also contributing to extracellular matrix homeostasis. Furthermore, we hypothesize that the upregulation of *Camp*, *S100a8*, and *S100a9* following SCF+G-CSF treatment may contribute to immune modulation in aged APP/PS1 mice (Figure 4).

Together with our previous findings, the present results suggest that SCF+G-CSF may promote synaptic repair and mitigate cognitive decline in AD (Figure 6). We further hypothesize that these therapeutic effects may be mediated, at least in part, through enhanced metal ion sequestration, which may reduce metal ion-associated neurotoxicity (Chen et al., 2023; Liu et al., 2022; Figures 4, 6 and Supplementary Figure S3), and through modulation of Rho GTPase-associated signaling pathways, including NADPH oxidase pathways involved in actin polymerization and synapse formation (Aguilar et al., 2017; Figure 6 and Supplementary Figure S3).

In addition, our findings highlight the potential value of investigating cancer-related pathways to improve our understanding of the molecular mechanisms underlying AD neuropathology. We found that *Cbl*, a gene implicated in cancer biology, was downregulated by SCF+G-CSF treatment and was represented in more than 40% of the enriched pathways identified by our pathway analyses (Figures 5, 6 and Supplementary Figure S3). Notably, four of the five most downregulated genes in SCF+G-CSF-treated APP/PS1 mice (*Cbl*, *Kcnq1ot1*, *Akap9*, and *Snhg11*) have been implicated in tumorigenesis or cancer progression (Figure 2B and Table 1). Cancer and neurodegeneration share overlapping biological features, including chronic inflammation, DNA damage, oxidative stress, and metabolic dysregulation (Driver et al., 2012; Houck et al., 2018). Epidemiological studies have also reported an inverse association between these two conditions, with individuals with AD exhibiting a lower risk of developing cancer and cancer survivors showing a reduced likelihood of developing AD (Driver et al., 2012).

Together with previous studies, our findings support the hypothesis that SCF+G-CSF may exert immunomodulatory effects, as reflected by the coordinated downregulation of several cancer-associated genes, particularly *Cbl*. Other cancer-associated genes, including *Kcnq1ot1* and *Akap9*, also showed reduced expression following SCF+G-CSF treatment. Although the functional significance of these transcriptional changes remains to be established, they suggest that molecular pathways shared between cancer and neurodegeneration warrant further investigation as potential therapeutic targets for AD.

Overall, our findings suggest that reducing AD pathology by broadly suppressing neuroinflammation may inadvertently impair beneficial microglial and innate immune functions. While some therapeutic approaches aim to suppress neuroinflammation or dampen immune responses in AD, our transcriptomic findings suggest that selected pro-inflammatory responses may be associated with enhanced microglial activation and innate immune function. Thus, our study suggests that the conventional neuroinflammatory model of AD may require further refinement and raises the possibility that effective therapeutic strategies should not only limit detrimental inflammation but also preserve or promote regulated immune responses that support amyloid clearance.

Figure 11 summarizes the diverse molecular pathways through which SCF+G-CSF may modulate AD-associated pathology. In addition to the classical hallmarks of AD, our findings highlight the potential contributions of metal ion dyshomeostasis, immune dysregulation, and molecular pathways shared with cancer biology. Together, these transcriptomic analyses provide new hypotheses and candidate molecular targets for future mechanistic studies investigating the therapeutic effects of SCF+G-CSF in aged APP/PS1 mice. Continued investigation of these interconnected biological processes may further improve our understanding of AD pathogenesis and facilitate the development of more effective therapeutic strategies.

**FIGURE 11:**
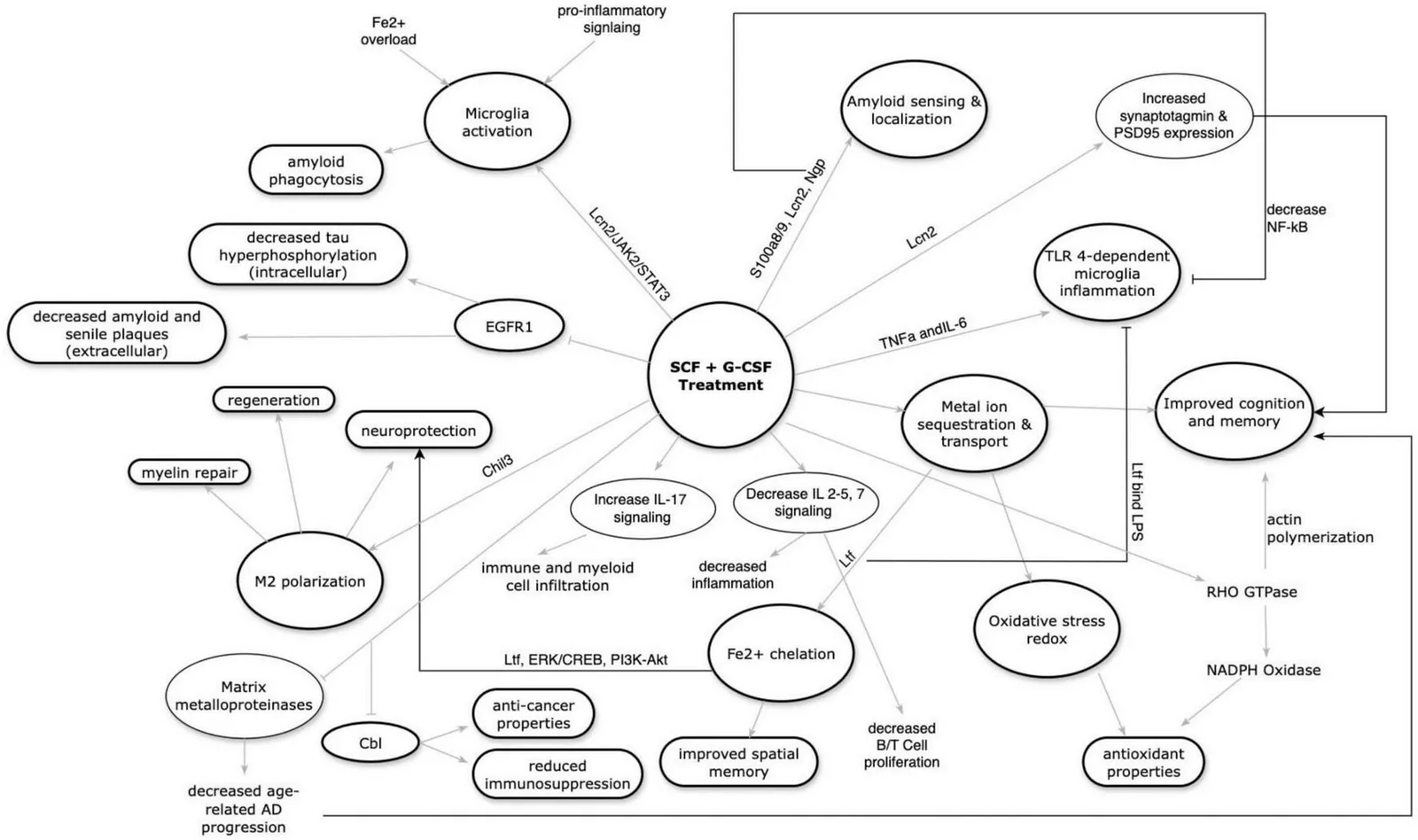
Proposed model of SCF+G-CSF-mediated molecular and cellular responses in aged APP/PS1 mice.

## Data Availability

The microarray data generated in this study have been deposited in the NCBI Gene Expression Omnibus (GEO) under accession number GSE345694 (https://www.ncbi.nlm.nih.gov/geo/query/acc.cgi?acc=GSE345694).

## Supplementary material

Supplementary Figure S1 Chromosomal analysis of SCF+G-CSF-induced differentially expressed genes (DEGs) using ShinyGO 0.77. **(A)** Analysis of SCF+G-CSF-induced DEG identifies a significantly higher proportion of protein-coding genes and a lower proportion of long non-coding RNA (lncRNA) genes than expected by chance. **(B)** Chromosomal mapping of SCF+G-CSF-induced DEGs shows that more genes are downregulated than upregulated across multiple chromosomes in aged APP/PS1 mice following SCF+G-CSF treatment.

Supplementary Figure S2 SCF+G-CSF treatment robustly increases *S100a8* and *S100a9* mRNA expression in the brains of aged APP/PS1 mice. qRT-PCR analysis of brain tissue from an independent cohort of aged female APP/PS1 mice (~28 months old) treated with vehicle or SCF+G-CSF for 5 consecutive days. Age-matched C57BL/6J wild-type (WT) mice served as controls. Brain tissue was collected immediately after the final SCF+G-CSF injection. Data are presented as mean ± SEM. WT, *n* = 3; APP/PS1, *n* = 5; APP/PS1 + SCF+G-CSF, *n* = 5. ****P* < 0.001; *****P* < 0.0001.

Supplementary Figure S3 Hypothesized immunomodulatory pathways associated with SCF+G-CSF treatment. **(A)** ShinyGO 0.77 analysis identifies a 29.1-fold enrichment of the IL-17 signaling pathway following SCF+G-CSF treatment, with 3 of 91 pathway-associated genes (*S100a8*, *S100a9*, and *Lcn2*) identified among the differentially expressed genes. **(B)** Enrichment of the IL-17 signaling pathway is further supported by STRING v12.0 analysis (see Figure 4). Based on the transcriptomic findings, we propose that SCF+G-CSF-induced enrichment of the IL-17 signaling pathway is associated with increased expression of *S100a8*, *S100a9*, and *Lcn2*, suggesting a potential immunomodulatory mechanism that warrants further investigation in aged APP/PS1 mice.

Supplementary Figure S4 SCF+G-CSF-treatment is associated with enrichment of metal sequestration and immune-related pathways in aged APP/PS1 mice. STRING v12.0 pathway enrichment analysis of differentially expressed genes in the brains of SCF+G-CSF-treated aged APP/PS1 mice, ranked by enrichment strength. **(A)** STRING v12.0 pathway enrichment analysis of SCF+G-CSF-induced differentially expressed genes identifies enrichment of pathways related to *metal sequestration by antimicrobial proteins*, innate immune responses, regulation of Toll-like receptors by endogenous ligands, IL-17 signaling, and the Toll-like receptor cascade. **(B)** Pie chart showing the relative distribution of the enriched pathways, organized by enrichment strength, illustrating the major functional categories represented in the transcriptomic dataset.

Supplementary Figure S5 Chromosomal distribution of differentially expressed genes in aged APP/PS1 mice following SCF+G-CSF treatment. Chromosomal mapping of differentially expressed genes (DEGs) using ShinyGO 0.77 demonstrates widespread distribution of DEGs across nearly all chromosomes in aged APP/PS1 mice receiving vehicle injections. In comparison, SCF+G-CSF treatment is associated with a markedly reduced number and altered chromosomal distribution of DEGs, suggesting attenuation of AD-associated transcriptional alterations. APP, aged APP/PS1 mice treated with SCF+G-CSF; CON, aged APP/PS1 mice injected with vehicle; WT, age-matched wild-type mice.

Supplementary Figure S6 ShinyGO 0.77 functional enrichment analysis identifies glycosaminoglycan degradation as the top-enriched pathway in aged APP/PS1 control mice. Functional enrichment was performed on 75 of the 89 differentially expressed genes (DEGs) identified in aged APP/PS1 mice receiving vehicle injections compared with age-matched WT mice. **(A)** Enriched pathways are ranked by fold enrichment, with glycosaminoglycan degradation identified as the most highly enriched pathway. **(B)** Interaction networks illustrating the functional relationships among enriched genes, including pathways associated with bacterial, viral, and parasitic infection.

Supplementary Table S1 Chromosomal localization of SCF+G-CSF-induced DEGs in the brains of aged APP/PS1. Start and stop represent the beginning and end of a gene length in base pairs. Table contents depicted in Supplementary Figure 5. Table from TAC.
